# Structure–activity modeling and hybrid machine learning-based prediction of bioactivity in pyrazole derivatives for drug discovery applications

**DOI:** 10.55730/1300-0152.2789

**Published:** 2025-12-25

**Authors:** Kader ŞAHİN, Serhat KILIÇARSLAN, Serdar DURDAĞI, Emin SARIPINAR

**Affiliations:** 1 Department of Medical Biochemistry, School of Medicine, Bandırma Onyedi Eylül University, Balıkesir, Turkiye; 2 Computational Biology and Molecular Simulations Laboratory, Department of Biophysics, School of Medicine, Bahçeşehir University, İstanbul, Turkiye; 3 Department of Software Engineering, Faculty of Engineering and Natural Sciences, Bandırma Onyedi Eylul University, Balıkesir, Turkiye; 4 Molecular Therapy Lab, Department of Pharmaceutical Chemistry, School of Pharmacy, Bahçeşehir University, İstanbul, Turkiye; 5 Computational Biology and Molecular Simulations Laboratory, Department of Biophysics, School of Medicine, Bahçeşehir University, İstanbul, Turkiye; 6 Laboratory for Innovative Drugs (Lab4IND), Computational Drug Design Center (HİTMER), Bahçeşehir University, İstanbul, Turkiye; 7 Systems Biology Lab, Biruni University Scientific Research Center, Biruni University, Istanbul, Turkiye; 8 Department of Chemistry, Faculty of Science, Erciyes University, Kayseri, Turkiye

**Keywords:** Pyrazole derivatives, 4D-QSAR, pharmacophore modeling, machine learning, drug design, GBM+RF hybrid

## Abstract

**Background/aim:**

Pyrazole derivatives are of growing interest due to their diverse pharmacological activities. However, their biological activity is often highly sensitive to subtle structural modifications. Existing quantitative structure–activity relationships (QSAR) approaches frequently fail to capture the conformational flexibility and nonlinear structure–activity relationships (SAR) of such heterocyclic scaffolds, creating a gap in the accurate prediction of their biological profiles. Therefore, there is a strong need for more robust and predictive computational frameworks. This study addresses this gap by integrating four-dimensional (4D)-QSAR descriptors with hybrid machine learning (ML) techniques to improve predictive accuracy and provide a more reliable tool for structure-based drug design. In this work, it was aimed to investigate the SAR of a series of pyrazole-based compounds using this advanced integrative computational strategy.

**Materials and methods:**

The dataset consisted of 54 pyrazole derivatives, of which 50 compounds were used for model construction and 4 compounds were reserved as a test set for validation. Although the test set was limited in size, the selected compounds were structurally representative of the training set, sharing the same core scaffold while covering different substitution patterns and biological activity values. The 4D-QSAR approach included multiple conformations of each compound and utilized matrix-based representations of geometric and electronic properties to capture dynamic molecular behavior. A pharmacophore model was generated using EMRE software based on the spatial and electronic features of used compounds. EMRE is an in-house software developed by our research group. It has been employed in several previously published 4D-QSAR studies for electron-conformational matrix of contiguity construction, pharmacophore modeling, descriptor matrix generation, and activity prediction. EMRE operates on standard geometric and electronic descriptors derived from quantum-chemical calculations, ensuring methodological transparency and reproducibility despite its proprietary implementation. Comparable performance trends obtained with EMRE-based 4D-QSAR models have been reported in previous studies, supporting the validity of the software for pharmacophore-driven QSAR analysis (Şahin et al., 2011; Sahin and Saripinar, 2020; Sahin et al., 2021).

Using this framework, a total of 204 molecular descriptors were computed using Spartan 07. To reduce redundancy and prevent overfitting, descriptor selection was optimized through a genetic algorithm (GA)-based procedure (Fernandez et al., 2011), and only statistically significant descriptors with low intercorrelation were retained for model construction. Subsequently, multiple ML algorithms, including artificial neural network, decision tree, and hybrid models, were evaluated to enhance prediction accuracy.

**Results:**

Among all the tested models, the gradient boosting machine and random forest (GBM+RF) hybrid algorithm yielded the highest predictive performance, with an R^2^ value of 0.99978. To assess the robustness of the ML models, the training and validation procedures were repeated using different random seed initializations. The resulting performance metrics showed only minor variations across runs, indicating that the predictive performance of the GBM, RF, and GBM+RF hybrid models was not sensitive to random seed selection. The overall dataset comprised 54 pyrazole derivatives, with 50 molecules used for model construction and 4 reserved for validation. Although the high R^2^ value indicates strong internal consistency, it should be interpreted with caution due to the relatively small sample sizes for the construction of the model and the test subset. Overall, the integration of 4D-QSAR and ML approaches demonstrated strong predictive capability and effectively captured the key geometric and electronic features associated with biological activity.

**Conclusion:**

The electron conformational-GA computational strategy provides a robust framework for the rational design and virtual screening of pyrazole derivatives, leveraging multi-conformer modeling and a diverse set of molecular descriptors to identify potentially active compounds. The study was limited by the small size of the training and test set and the absence of experimental validation, which may constrain the generalizability of the findings. Future work could address these limitations by applying the model to larger and more diverse ligand dataset, performing virtual screening of compound libraries to discover novel hits, and validating promising candidates through in vitro assays. Overall, these findings support the potential of this scaffold for drug discovery, while further experimental studies are warranted to confirm the predicted activities and refine the predictive power of the model.

## 1. Introduction

Pyrazole derivatives have long attracted attention in medicinal chemistry, owing to their remarkable pharmacological versatility. A recent mini review emphasized that pyrazole-based compounds display a wide spectrum of bioactivities, including antidiabetic, antimicrobial, antiparasitic, antiviral, and anticancer effects—underscoring the pyrazole ring as a highly privileged scaffold in drug discovery (Ahmed et al., 2024). In fact, many marketed drugs and lead compounds across diverse therapeutic areas, such as nonsteroidal antiinflammatories (e.g., celecoxib) and central nervous system (CNS) agents, incorporate the pyrazole nucleus, highlighting its proven pharmacological value.

The pyrazole scaffold represents a class of potent checkpoint kinase 1 (Chk1) inhibitors that target the serine/threonine protein kinases involved in DNA damage response mechanisms (Tao et al., 2007). Chk1 plays a pivotal role in the regulation of DNA damage checkpoints and has emerged as a promising target for cancer therapy. Additionally, tyrosine kinases from the Src family, comprising homologous proteins such as Src, Yes, Fyn, Lyn, Hck, Blk, Brk, Fgr, Frk, Srm, Lck, and Yrk, have been implicated in various pathological conditions (Zhai et al., 2021; Luo et al., 2022). Inhibitors of Src enzymes have been observed to play a significant role in myocardial infarction, stroke, osteoporosis, and tumor progression, particularly in breast, ovarian, and pancreatic cancers (Noronha et al., 2007). The current study focuses on exploring pyrazole-based derivatives for their anticancer potential and aimed to design novel pyrazole compounds with improved biological activity.

The conventional approach to drug design is widely recognized as inconvenient, interdisciplinary, costly, and time-consuming. According to recent data released by the National Center for Science and Engineering Statistics, the expenditure on research and development (R&D) in the United States amounted to $666.9 billion in 2019 and increased to $708.0 billion in 2020, reflecting a continuous upward trend (National Patterns of R&D Resources^1^, 2019–20). These figures underscore the growing need for efficient computational strategies capable of predicting biological activities of hit compounds at early stages. Consequently, the ability to estimate activity-related characteristics from structural data has become a critical challenge in modern drug discovery. Numerous approaches in drug design rely on establishing quantitative connections between molecular structure and biological activity, commonly referred as ligand-based approaches or quantitative structure–activity relationships (QSAR) studies.

However, despite abundant data, establishing reliable structure–activity relationships (SAR) for pyrazole derivatives remains challenging (Karali et al., 2007). Their biological activity is exceptionally sensitive to subtle structural and stereochemical modifications, which can dramatically alter potency and selectivity. Traditional QSAR and three-dimensional (3D)-QSAR models often fall short in these contexts because they typically rely on a single or limited set of molecular conformations and neglect the dynamic flexibility that characterizes small heterocyclic compounds (Muratov et al., 2020). QSAR models nevertheless play a crucial role in elucidating ligand–biological activity relationships and guiding the design of novel compounds with specific biological properties (Martins et al., 2009). In QSAR studies, molecular structures are represented by a set of descriptors whose numerical values significantly influence biological activity (Bernazzani et al., 2006), enabling the identification of key structure–activity patterns that support rational drug design.

QSAR models reveal mathematical relationships between chemical, biological, physical, and environmental factors, using computable variables such as electronic, physicochemical, topological, and stereochemical indices (Melagraki et al., 2006). Numerous successful applications of SAR and QSAR models have been reported, highlighting their effectiveness in diverse drug discovery contexts (Patel and Bhatt, 2021; Kim and Jeong, 2022; Chen et al., 2023a).

A more detailed approach, known as 3D-QSAR, incorporates the spatial characteristics of molecular structures and provides insights into how molecular shape and spatial feature distribution influence biological activity. Building on this framework, 4D-QSAR has emerged as a valuable extension, particularly when receptor structures are well-defined (Romeiro et al., 2005). Unlike traditional 3D-QSAR, 4D-QSAR introduces an additional dimension that accounts for multiple conformations, incorporating constructional knowledge for a group of conformers rather than relying on a single static structure (Duca and Hopfinger, 2001; Andrade et al., 2010). This approach captures conformational variability and diversity and enables a more realistic representation of molecular flexibility and stability, which are critical determinants of bioactivity in flexible molecules (Becker et al., 2000).

To address conformational limitations, 4D-QSAR methods have been developed to incorporate conformational ensemble averaging, enabling a more realistic representation of dynamics of compounds and conformational space across multiple alignments and sampling (Li et al., 2025). Nevertheless, even 4D-QSAR models alone may lack sufficient predictive power when applied to large, chemically diverse datasets or when modeling complex nonlinear relationships between structure and activity.

In 4D-QSAR, the generation of multiple conformers allows for a detailed exploration of conformational space and molecular flexibility (Şahin et al., 2011). Through molecular dynamics simulations, shared pharmacophoric arrangements and recurring 3D motifs among active compounds can be identified, offering valuable insights into the structural determinants of biological activity (Krasowski et al., 2002; Hong and Hopfinger, 2003).

The electron-conformational method (ECM) operates on the premise that both electronic and conformational features play a decisive role in determining biological activity (Rosines et al., 2001). Within the ECM framework, molecules are characterized using electronic and conformational descriptors that quantitatively represent key structural attributes influencing bioactivity. In parallel, pharmacophore concepts provide a 3D map of essential features shared among active conformations, guiding rational compound design (Seidel et al., 2020). Advanced approaches such as the electron-topological method (ETM) and electron-conformational (EC)-based pharmacophore-finding techniques further enhance the identification of bioactivity-relevant structural features (Dimoglo et al., 1995; Bersuker et al., 1999a).

QSAR modeling requires quantified biological activity data, molecular descriptors, and appropriate statistical or machine learning (ML) methods to establish predictive relationships. Various optimization and feature-selection techniques, including genetic algorithm (GA) (Devi et al., 2021), principal component analysis (PCA), partial least square (PLS) (Liang et al., 2021), and neural network (Xu et al., 2018), have been widely employed to improve model performance and interpretability.

In recent years, ML has emerged as a transformative approach in cheminformatics and drug design, particularly for large and high-dimensional datasets where classical statistical methods are insufficient (Rodríguez-Pérez et al., 2022). By effectively capturing complex and nonlinear relationships between molecular descriptors and biological activity, ML algorithms have enabled the development of highly predictive QSAR models. Recent reviews further highlight that integrating ML algorithms with QSAR descriptors significantly enhances predictive accuracy and facilitates the exploration of complex chemical spaces (Niazi and Mariam, 2023). Moreover, hybrid ML strategies improve model robustness and scalability, offering compelling advantages for virtual screening and lead optimization while reducing the time and resource demands of conventional experimental approaches (Koirala et al., 2025).

Given these developments, there remains a clear unmet need for robust and integrative computational frameworks that combine the conformational richness (Son et al., 2012) of 4D-QSAR with the predictive strength of ML, especially for privileged scaffolds such as pyrazole, where minor structural modifications can have pronounced effects on activity.

In this study, the EC-GA technique was introduced as a hybrid approach that integrates GA optimization with the EC method to identify key structural parameters associated with receptor binding affinity and pharmacophore elucidation. In addition, a tailored hybrid ML model combining gradient boosting machine (GBM) and random forest (RF) algorithms was developed, incorporating recursive feature elimination (RFE) to capture intricate nonlinear relationships between molecular descriptors and biological activity. The proposed framework aimed to enhance predictive accuracy and provide deeper insights into the SAR of pyrazole derivatives. The study focuses on a dataset of 54 pyrazole compounds reported by Tao et al. (2007), representing a chemically diverse series suitable for comprehensive SAR analysis.

## 2. Materials and methods

The computational workflow applied in this study followed a stepwise and reproducible pipeline: 1) selection of pyrazole derivatives from a single experimental source, 2) quantum-chemical descriptor calculation using Spartan 0.7 (2007, Wavefunction Inc.)^2^, 3) construction of 4D-QSAR pharmacophore models using EMRE software, 4) GA-based descriptor selection, 5) ML model training using GBM, RF, and a the GBM+RF hybrid framework, and 6) model validation using internal cross-validation and a test set.

### 2.1. Dataset and molecular structure preparation

A total of 54 pyrazole derivatives were obtained from the study by Tao et al. (2007). All compounds were manually drawn and processed in Spartan 0.7, which was used for geometry optimization, conformer generation, energy minimization, and descriptor calculation. Each structure was optimized using the MMFF94 force field and the lowest-energy conformer was selected for further analysis. A full list of the 54 compounds, including their 2D structures and both experimental and calculated half-maximal inhibitory concentration (IC_50_) values, is provided in Table 1 and Table S1 to ensure reproducibility. As summarized in Table 1, the dataset spans a broad range of pIC_50_ values, with highly active compounds clustered around substitutions at the C-3 and C-5 positions.

### 2.2. Modeling and 4D-QSAR

The investigation of the interaction mechanism between a ligand molecule and a receptor in biorecognition is directly related to the biological activity of the studied molecule and interaction with the bioreceptor. As this interaction mechanism is highly complex, it poses one of the most challenging problems in biochemistry. Information about receptors is generally limited, and their interactions with small molecules are complicated due to external factors such as metabolism. This can be addressed by resorting to various biological methods and physicochemical techniques at each stage of the ligand–receptor interaction. However, in general, this approach is lengthy and challenging to resolve. The most challenging aspect of the QSAR methods employed is the inability to clearly elucidate the interaction mechanism between the receptor and the drug-like small molecule. Therefore, the interaction mechanism of the ligand–receptor can be likened to a closed box with unknown contents. The specificity of the inputs to be examined is low because it is not clear which type of molecular properties will be used in the formula for biological activity. In many QSAR approaches, molecular modeling methods have been utilized in computer calculations (such as topological indices, molecular graphs, Cartesian coordinates, atomic charges, bond orders, bond lengths, etc.) (Oprea et al., 1994; Tokarski and Hopfinger, 1997).

QSAR models play a crucial role in elucidating the mechanisms of effects of used ligands in training set and facilitate the enhancement and design of innovative compounds with specific biological attributes (Martins et al., 2009). QSAR studies involve the characterization of biological activity of compounds by considering their diverse structural features, highlighting how variations in chemical composition contribute to biodiversity. In drug design, the goal is to establish a mathematical relationship between molecular features and the biological activity of a compound. The structural attributes of molecules are commonly represented by a set of physicochemical parameters, and the values of these parameters influence the activity of molecules in a specific manner (Bernazzani et al., 2006).

### 2.3. EC-GA method

The ETM (Saripinar et al., 1996) is a structure–activity analysis approach that relates biological activity to electronic and geometric features of molecules using 3D electron topological compatibility matrices derived from a single optimized conformer. Although ETM enables the identification of pharmacophoric features based on electronic descriptors, its reliance on a single conformer and binary activity classification represents a major limitation for flexible molecules. To overcome these drawbacks, the ECM was employed in this study. ECM extends the ETM framework by explicitly considering all energetically accessible conformers of each compound and identifying a common pharmacophoric pattern across the entire series, thereby providing a more realistic description of ligand flexibility and SAR. In the present work, ECM was further combined with a GA to optimize descriptor selection and enhance model robustness, allowing the systematic elimination of less informative parameters and improving predictive performance compared to classical ETM-based approaches.

The current study focuses on investigating the relationships between the structure of pyrazole derivatives and their anticancer activity. The EC-GA method was employed for this analysis. The biological activity data were acquired from existing literature sources 1. In the QSAR and pharmacophore modeling, the logarithm of quantified IC_50_ (nM) values was employed to establish a linear correlation with free energy change. Transformation to the logarithmic scale for IC_50_ values was implemented to reduce potential errors in the analysis. The 3D configurations of the molecules were generated using Spartan 0.7 software, and subsequent energy minimization was performed through MMFF94 molecular mechanics. Subsequently, a comprehensive optimization of the structures was conducted using the semiempirical Parametric Method 3 (PM3) level theory. Quantum-chemical semiempirical PM3 calculations were performed for a total of 54 resulting molecules. The conformational arrangements of all the compounds were explored using molecular mechanics, and conformational assessment was conducted at the PM3 level using the randomized search technique employed by Monte Carlo (Hehre, 2003). Following this, conformers with imaginary frequencies were eliminated. The presence of an imaginary frequency indicates a transitional state for the molecule and implies that the optimization is not complete (Librando and Alparone, 2009). Herein, the study was initiated by optimizing molecular structures using Spartan 0.7. Subsequently, Spartan 0.7 files were seamlessly transferred to EMRE software, renowned for its capability to extract and compute a spectrum of parameters critical for QSAR investigations. EMRE excels in deriving topological, geometrical, constitutional, electrostatic, and quantum chemical parameters, making it a prevalent choice in numerous QSAR studies.

**Figure 1:**
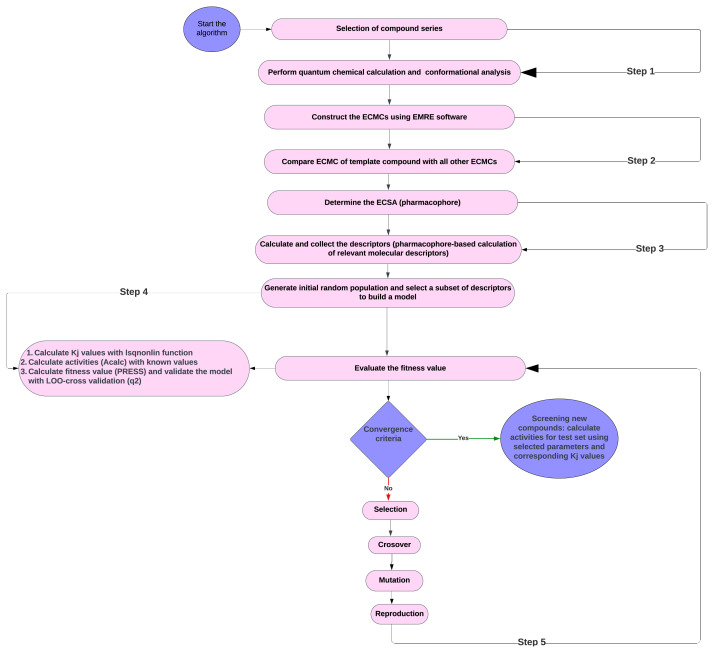
Schematic representation of the proposed hybrid evolutionary computation–GA method. The workflow illustrates the main steps of the method, including 1) initialization of the population, 2) evaluation of candidate solutions using the fitness function, 3) selection of high-performing candidates, 4) application of crossover and mutation operators to generate offspring, and 5) iterative optimization until convergence criteria were met. Key decision points, feedback loops, and data flow between modules are indicated to facilitate understanding of the hybrid algorithm.

The dataset for the 4D-QSAR investigations, along with the corresponding predicted biological activities, is presented in Table 1. The study comprised a total of 54 compounds, with 50 compounds allocated to the training set for model development and 4 compounds labeled for the test set to validate the predictive capacity of the model. To ensure a robust and unbiased representation, the selection of compounds for both the training and test sets was executed through a randomized process. This approach underscores our commitment to a comprehensive exploration of molecular properties and their correlation with biological activities, leveraging advanced computational tools and rigorous methodology. The fundamental stages of the EC-GA study are illustrated in Figure 1.

A training set comprising compounds with well-established bioactivity was selected. Then, electronic structure calculations were conducted and the 3D geometry for all the molecular entities was optimized. The geometry optimization yielded a conformational ensemble with an energy threshold of 1.5 kcal/mol, generating a maximum of 100 conformers per molecule. Recognizing the flexibilities of the collected molecules, each compound was treated as an accumulation of conformers representing distinct regions in conformational space. For every compound in the series, n × n matrices, denoted as the electron-conformational matrix of contiguity (ECMC), were constructed from the output files, where n represents the atom count in the compound. ECMC incorporated both geometric and electronic characteristics, aiming to comprehensively capture the features of the molecules under investigation (Bersuker et al., 1999b).

The diagonal component, a_ii_, within the ECMC corresponded to various electronic atomic characteristics linked with the *i*^th^ atom in the compound. These characteristics included valence values, atomic charges, atomic polarizability values, highest occupied molecular orbital (HOMO), and lowest unoccupied molecular orbital (LUMO) energies. The index, i, in this context, represented the *i*^th^ atom in the compound. The off-diagonal component, a_ij,_ took on two distinct types. For bonded atoms, represented by i and j, a_ij_ encompassed electronic parameters such as the Wiberg index, bond energy (ionic, covalent, or total), bond order, and bond polarizability. Conversely, when i and j denoted nonbonded atoms, a_ij_ signified the distance between the *i*^th^ and *j*^th^ atoms. An ECMC was generated for each compound in the series. To discern the matrix components, present in all active compounds but absent in inactive compounds, the ECMC was employed as a model for one of the active compounds. It was then subsequently compared, within specific tolerance limits, with the ECMCs of other compounds. The recognized submatrix was denoted as the contiguity for activity (EC submatrix of activity (ECSA)) EC submatrix (Marenich et al., 2008). Consequently, the Pha, known as the activity property, was defined as a submatrix encompassing both interatomic distances and structural electron descriptors. In the EC-GA framework employed herein, pharmacophoric features were identified algorithmically as ECSA submatrices rather than as classical 3D graphical pharmacophore alignments. This matrix-based representation encodes the essential electronic and geometric features responsible for biological activity. After pinpointing the ECSA with a reduced number of matrix components, evaluating the flexibility of each descriptor becomes a straightforward task. In instances where the identified submatrices are not adequately replaced, adjusting the tolerance limits until appropriate fragments are identified is recommended. If the obtained properties yield compelling results, the ECSA can be conveniently assessed for activity. However, if not, a more in-depth investigation into the deficiency of the activity feature should be conducted, and the entire process should be repeated.

The IC_50_ values (in nM) were first converted to molar units, and then transformed into negative logarithmic form of IC_50_ (pIC_50_). Compounds exhibiting pIC_50_ ≥ 7.61 were classified as high-activity compounds (n = 28), whereas those with pIC_50_ < 7.61 were labeled as low-activity compounds (n = 26) during the initial Pha atom classification. The derivation of Pha groups involves comparing a template active compound with the rest of the compound series using weighted graphs. For each selected model compound (either low-active or high-active), its ECMC is compared with those of the other compounds. Constraints on flexibility play a crucial role in optimizing the Pha return. For example, expanding the upper limit of any submatrix component beyond a certain point will result in weakly active compounds exhibiting high activity characteristics (Altun et al., 2003). Subsequently, peak tolerance values for all conformers of the compounds were calculated without restrictions on tolerance values, and ECSA were obtained. As part of enhancing the 4D-QSAR modality, a significant task is to develop the most effective activity estimation function. It is noteworthy that the most active conformer is not always the one with the lowest energy, necessitating the consideration of the compounds’ flexibility. Considering the potential impact of minor energy differences between conformers on electronic structure diversity, it became essential to incorporate Boltzmann populations and dynamics for all compounds within the 4D-QSAR strategy (Santos-Filho and Hopfinger, 2006). This inclusive approach is critical for gaining insights into the effects on the biological activity of all conformers that exhibit stability in energy (Pavlov et al., 2007).

The final bioactivity assessment formula in Eq. (1) incorporated not only the lowest energy conformation but also all the viable conformers listed in Table S1. The contributions of each compound conformation were considered through the Boltzmann distribution. Table S2 represents the relative energies and Boltzmann values for 64 conformers of the most active compound **27** in the pyrazole series. Eq. (1) incorporates the general activity formula proposed by Bersuker (2003, 2008):


(1) $$A_{n}=A_{0}\frac{\sum_{i=1}^{me}\delta_{ni}[Pha]e^{-S_{ni}}e^{-E_{ni}/kT}}{\sum_{i=1}^{me}e^{-E_{ni}/kT}}$$

here, δ is the Kronecker delta, a function of two parameters that is 1 if the pharmacophore exists and 0 if not. A_n_ and A_l_ represent the activities of the *n*^th^ compound and the reference molecule, respectively, and m_n_ is the number of conformations of the *n*^th^ molecule. E_li_ is the relative energy of the *i*^th^ conformer of the reference compound, E_ni_ is the relative energy of the *i*^th^ conformer of the *n*^th^ compound (in kcal mol^−1^), R is the gas constant (in kcal mol^−1^ K^−1^), and T is the temperature in Kelvin.

Auxiliary group (AG) and antipharmacophore shielding (APS) exerted their influence on the activity of all molecular structures under consideration. This variability is why certain compounds, featuring a pharmacophore, showed varying effectiveness. Consequently, it became viable to characterize APS and AG as molecular descriptors. The impact of these descriptors was quantified as the sum of all these factors, utilizing the feature S in the following manner:


(2) $$S_{ni}=\sum_{j=1}^{N}\kappa_{j}a_{ni}^{j}$$

here, in the *i*^th^ conformer of the *n*^th^ molecule, (a_ni_^(j)^) represents the parameter defining the *j*^th^ type of impact, with N being the number of chosen parameters. The activity exhibited exponential dependence on S (A e^S^). Thus, it was assumed that S encapsulated specific properties of the ligand–receptor interaction, facilitating quantitative activity detection. The adjustable (variational) constants (κ_j_) were computed using the function S, considering the dependence of the Boltzmann population on the energy and temperature of each conformation. The *lsqnonlin* function in the MATLAB statistics toolbox^3^ was employed to numerically solve the scheme of differential equations for the highest parameter subset, obtaining values for the respective model parameters. These parameters captured specific molecular structure characteristics and were typically categorized into parameter classes based on the represented data (Todeschini and Consonni, 2000), (Figure 1). The fundamental assumption of QSAR modeling is that there exists an association between molecular structure and physical or biological characteristics. The primary necessity involves employing a technique to encode diverse structural characteristics within a molecule. This requirement is fulfilled by molecular parameters, which are numerical representations, typically, of specific submolecular features. A total of 204 parameters (Table S3) were computed, falling into four categories: a) electronic, b) thermodynamic, c) geometrical, and d) quantum-chemical. In terms of their 3D coordinates, geometric parameters served to characterize the shape and size of the molecule. Precise coordinates were essential for these parameters, requiring the structure to undergo geometry optimization before calculation. Examples include inertia moment (Goldstein et al., 2001), molecular surface area and volumes (Zhang et al., 2017), and parameters of shadow.

Electronic parameters encompass a range of characteristics pertaining to the molecular electronic environment. These include the energies of the HOMO and LUMO, as well as electronegativity and various partial charge attributes focused on specific atoms. Quantum chemical parameters, derived directly from the orbital energies of optimized geometries, consist of hardness, chemical potential, ionization potential, electron affinity, and electrophilicity (Padmanabhan et al., 2007). Thermodynamic parameters aimed to define the condition of the system, categorized into two fundamental groups: extensive attributes and intensive characteristics. Extensive parameters vary with the system and encompass entropy, enthalpy, mass, volume, and internal energy. Conversely, intensive parameters, such as pressure, temperature, chemical potential, and Gibbs free energy, remained independent of the system’s scale. The GA technique was utilized for the identification of the most pertinent parameters. The objective of this method is to pinpoint the minimum set of relevant structural parameters required for predicting biological activity. Optimization poses a primary challenge in QSAR modeling, and this content covers two noteworthy applications of optimization methods. The initial application involved fine-tuning the parameters of a model within a predefined neural feedforward network, including biases and weights. Another critical aspect where optimization played a pivotal role was in the process of selecting parameters.

In this context, the selection of compounds from a library or library design can be framed as choosing from distinct products (Gillet et al., 2002), or the process of selecting parameters to construct models can be expressed. GA belongs to the category of algorithms known as evolutionary algorithms (Revard et al., 2014), leveraging concepts from biological evolution to derive efficient solutions for optimization problems. In the domains of QSAR modeling (Guha and Jurs, 2004), cheminformatics (Venkatraman et al., 2004), and chemometrics (Hervás et al., 2004), GA approaches have been widely employed. This study emphasizes the application of GA approaches to explore high-dimensional spaces as effective tools. Specifically, one implementation of GA in QSAR modeling is to explore a parameter space to identify optimal subsets of parameters that can be utilized to create selective models. GA is a simulation method that mimics certain procedures observed in natural evolution and have been effectively employed in QSAR analysis for the selection of relevant characteristics.

In the current study, initial data reduction was carried out using GA in MATLAB software (version 7.0) with the following configurations: number of iterations, 500; number of generations, 500; population size, 500; probability of mutation, 0.01; and probability of cross-over, 0.5. QSAR models, built with 12 descriptors following the 5:1 guideline, were established. The inherent significance of each descriptor was provided. The fitness function plays a pivotal role in gauging the convergence speed of a GA process. In the current methodology, the predictive remaining sum of squares (PRESS) (Eq. (3)) was employed as the fitness criterion. In this investigation, the fitness value for each descriptor was computed through leave-one-out cross-validation (LOO-CV) (Consonni et al., 2009). For a multitude of valid examples n, PRESS serves as a benchmark index for evaluating the precision of a modeling method based on the LOO-CV technique. PRESS is defined as the summation of the squared differences between the experimental (exp) values and the calculated (calc) values.


(3) $$PRESS_{N}=\sum_{n=1}^{N}|A_{n}^{exp}-A_{n}^{calc}{|}^{2}$$

Here, the experimental activity (A_n_*^exp^*) represents the observed values, while A_n_*^calc^* signifies the activity predicted by the LOO-CV model, utilizing N parameters (where, in this context, N equals 12). The cross-validated correlation coefficient q^2^ serves as a metric quantifying the predictive performance of the model. It was computed by assessing the disparities between the experimental activity and the model-predicted activity, as shown in Eq. (4):


(4) $$q^{2}=1-\frac{\sum_{n=1}^{N}|A_{n}^{exp}-A_{n}^{calc}{|}^{2}}{\sum_{n=1}^{N}|A_{n}^{exp}-{\bar{\text{A}}}_{n}^{exp}{|}^{2}}\equiv 1-\frac{PRESS}{ssy}$$

Here, N represents the total number of training set compounds within the complete dataset. SSY corresponds to the sum of the squares of deviations between the experimental values (A_n_*^exp^*) and their mean. (A_n_*^exp^*) signifies the measured values of the dependent parameter, and Ā_n_*^exp^* represents the average value of the dependent parameter calculated over the entire training set. A low PRESS suggests a high level of predictability within the model. SSY represents the sum of squares linked to relevant sources of variation. Typically, a q^2^ value exceeding 0.5 is commonly regarded as evidence of the model’s strong predictive capability.

This criterion is frequently employed by software such as CoMFA, marketed by Tripos^4^ as a potential indicator of a model’s predictive efficacy. If q^2^ is low, it may suggest that the model has limited predictive capability, but the opposite is not necessarily true (Golbraikh and Tropsha, 2002). Evaluating the predictive powers of a QSAR model involves its ability to accurately estimate the target property of molecules not included in the model’s development. Consequently, to substantiate the predictive power of a model, one essential criterion is to partition the compounds within a series into training and test sets. Following the development of a model on the training set, its effectiveness was assessed by its precision in predicting the target property of the test set (Gramatica et al., 2004).

In this study, the EC-GA methods were devised through a random combination of compounds for both the test and training sets. Multiple methods were established, each characterized by R^2^ (cross-validation, cv) and R^2^ (noncross-validation, ncv) values. As emphasized by Golbraikh and Tropsha (2002), while high R^2^ (cv) values are crucial, they alone are insufficient to determine the credibility of a given model. To address this, they advocated for the inclusion of an external set to validate the reliability and robustness of a model. Consequently, the predictive capabilities of all constructed models were evaluated by computing their R^2^ using the corresponding test set compounds (Van Damme and Bultinck, 2007). Two distinct equations, previously discussed by Schuuremann et al. (2008), were employed for the calculation of q^2^ from an external evaluation set:


(5) $$q_{ext1}^{2}=1-\frac{\sum_{n=1}^{N}|A_{ntest}^{exp}-A_{ntest}^{calc}{|}^{2}}{\sum_{n=1}^{N}|A_{ntest}^{exp}-\;{{\bar{\text{A}}}_{tr}^{\exp}}^{2}}$$

(6) $$q_{ext2}^{2}=1-\frac{\sum_{n=1}^{N}|A_{ntest}^{exp}-A_{ntest}^{calc}{|}^{2}}{\sum_{n=1}^{N}|A_{ntest}^{exp}-{\bar{\text{A}}}_{ntest}^{exp}{|}^{2}}$$

In the model construction, A_n_*^exp^* refers to the experimental activity, A_n_*^calc^* corresponds to the predicted activity, N signifies the count of tested molecules excluding the compound omitted during the model development, and Ā_n_*^exp^* stands for the average of experimental activities.

### 2.4. Proposed GBM+RF hybrid model

A total of 273 molecular descriptors, including topological descriptors, QikProp-derived physicochemical properties, and semiempirical quantum chemical parameters, were calculated using the Molecular Descriptors module of the Schrödinger software^5^ Suite. These descriptors were subsequently utilized as input features for ML algorithms to predict the biological activity of pyrazole derivatives. This approach enabled the development of data-driven predictive models that captured both structural and electronic characteristics relevant to bioactivity.

In this study, various artificial intelligence (AI) algorithms were used to compare model performance in data analysis and prediction tasks. The pyrazole derivatives were subjected to different ML algorithms to model and predict their biological activity. Artificial neural network (ANN), GBM, logistic regression (LR), decision trees (DT), and RF were the methods employed. Each algorithm symbolizes a unique learning approach and has its own merits in capturing SAR. ANN models can learn highly nonlinear and complex patterns due to the multilayered architectures. The GBM, being a sequential ensemble method, reduces the residual errors iteratively and thus is very good to model even the slightest nonlinear interactions. LR was used as a reference model to evaluate linear relationships and to give a comparison point. DT models divide the feature space into understandable hierarchical decision rules, while RF being an ensemble of many decorrelated DT diminishes variance and increases robustness. Thus, even with small or noisy datasets, the results will be accurate. The use of such a diverse range of algorithms allowed for their predictive behavior to be assessed comprehensively and the most reliable components for hybrid modeling to be identified.

Moreover, the complementary strengths of GB and RF inspired the idea of the proposed GBM+RF hybrid framework, which proved to have better generalization performance than single models. These algorithms were tested both individually and, in some cases, as part of hybrid models combining multiple techniques.

The hybrid modeling approach aimed to enhance generalizability and reduce error rates by leveraging the strengths of different algorithms. Since each method performs differently depending on the structure and features of the data, this study evaluated the diversity of approaches and compared their performance to identify the most effective ones.

RF is an ensemble learning method where multiple DT work together to produce strong predictive results (Breiman, 2001). It is widely used for both classification and regression problems due to its high accuracy, low variance, and strong generalization capability. The algorithm is based on the principle of bagging, where numerous independent DT are built using randomly selected bootstrap samples from the training data. Each tree is trained not only on a random subset of the data but also on a random subset of features, which increases model diversity and helps reduce the risk of overfitting. The prediction process is straightforward; each tree makes an independent prediction, and these predictions are aggregated to produce the final output. For regression problems, the final prediction is calculated by taking the arithmetic mean of the outputs from all trees:


(7) $$\hat{y}(x)=\frac{1}{M}\sum_{m=1}^{M}T_{m}(x).$$

Here, ỹ(*x*) represents the final prediction made by the model for observation *x*, *Tm*(*x*) denotes the prediction made by the *m*^th^ DT for the same observation, and *M* indicates the total number of trees in the model.

RF models are particularly effective when working with high-dimensional datasets, missing values, or noisy data. They also demonstrate strong tolerance for complex intervariable relationships. One of the key advantages of this method is its interpretability. Due to the feature importance scores, it is possible to assess how much each variable contributes to the overall predictive performance of the model.

GBM (Friedman, 2001) is a powerful prediction algorithm that falls under the category of ensemble learning methods. It works by sequentially training weak learners typically DT where each new model focuses on correcting the errors made by the previous one. Due to this iterative learning structure, GBM can deliver high predictive accuracy, especially in datasets with nonlinear and complex relationships. The GBM algorithm used in this study was based on a sequential learning strategy that minimizes prediction errors iteratively. The model construction process can be described in three main mathematical steps: initial prediction, error estimation based on the negative gradient, and model update. These steps are detailed below along with their respective equations: In the initial prediction (Eq. (8)), the first step involved determining a constant initial value for the entire dataset. This value was typically the constant score that minimized the chosen loss function:


(8) $$F_{0}(x)=\arg\;\underset{c}{\text{min}}\sum_{i=1}^{n}L(y_{i},c).$$

Here, L ( *yi,c*) represents the loss between the true value, *yi*, and the constant prediction, *c*. This forms the base prediction in the zeroth iteration of the model.

### 2.5. Error estimation – negative gradient

In the second step, the negative gradient of the loss function with respect to the predictions was calculated for each observation. These residuals served as the target values for training the next weak learner.


(9) $$r_{i}^{\left(m\right)}=-{\left[\frac{\partial L(y_{i},F(x_{i}))}{\partial F(x_{i})}\right]}_{F=F_{m}-1}$$

Here, *ri*^(m)^ denotes the negative gradient computed for the *i*^th^ observation at the *m*^th^ iteration.

### 2.6. Model update

Finally, the model output was updated by adding the output of the new learner to the previous model prediction, scaled by a learning rate, η:


(10) $$F_{m}(x)=F_{m-1}(x)+\eta .h_{m}(x).$$

Here, *Fm*(*x*) is the updated prediction at iteration m, *hm*(*x*) is the output from the new learner, and *n* is the learning rate.

These three steps were repeated iteratively. With each step, the model reduced the prediction errors, gradually improving its accuracy. This sequential structure enabled GBM to perform exceptionally well, particularly on datasets with nonlinear and complex relationships.

In this study, a hybrid model was developed by combining two tree-based ensemble learning algorithms: GBM and RF. These two algorithms complement each other in terms of their learning approaches. GBM builds new trees that focus on correcting the errors made by previous predictions, thereby incrementally improving the model’s accuracy. This sequential optimization process allows GBM to perform exceptionally well, especially in datasets with complex and nonlinear relationships. On the other hand, RF uses a bagging approach, combining the results of numerous DT trained independently on random subsets of the data. This structure enhances the model’s generalization ability and provides strong resistance to overfitting. RF is also known for producing stable predictions even with high-dimensional and noisy data.

**Figure 2:**
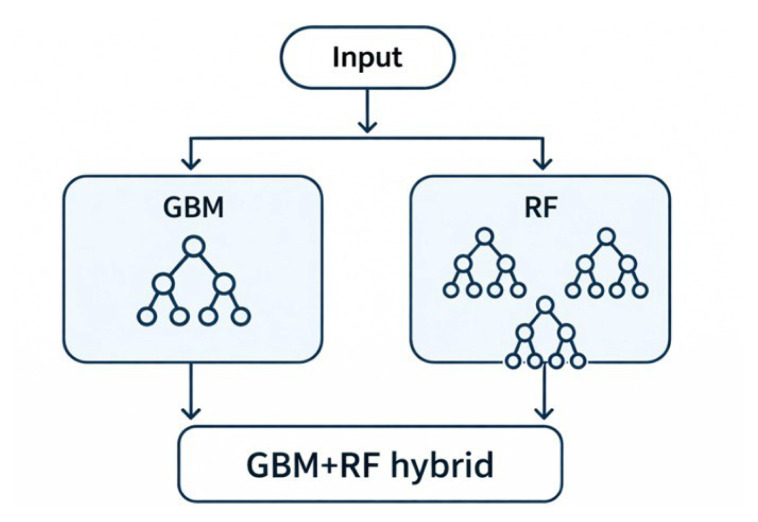
Schematic overview of the proposed GBM+RF hybrid model. The figure illustrates the integration of GBM and RF algorithms in a hybrid framework. Input features are first preprocessed and normalized before being fed into both models. GBM captures complex nonlinear patterns through sequential tree boosting, while RF aggregates predictions from multiple DTs to enhance robustness. The outputs of both models are combined using a weighted ensemble strategy to generate the final prediction. Key components, including feature selection, model training, cross-validation, and final ensemble prediction, are highlighted to facilitate understanding of the hybrid approach.

In the proposed hybrid model, GBM was first used to learn the relationships between structural variables and the target variable. The output scores generated by GBM were then used as input for the RF model to produce the final predictions. By combining GBM’s strength in error reduction with RF’s power in reducing variance, the resulting model achieved higher accuracy and better generalizability. This approach offers an effective solution for challenges like high dimensionality, multicollinearity, and data scarcity, which are common in the prediction of biological activity in chemical compounds (Figure 2).

The whole process of the proposed GLM+RF hybrid model is expressed as a single function, as shown in Eq. (11):


(11) $$\hat{y}(x)=\frac{1}{M}\sum_{m=1}^{M}T_{m}(x).$$

The hybrid model that was proposed was built using a two-stage stacked structure in which the RF model acted as a meta-learner on the output scores from the GBM. The model was designed this way to take advantage of the complementary strengths of both ensemble algorithms. By carefully correcting and modeling complexities in the descriptor space, GBM reduces the bias of the model effectively. However, models based on GBM can have high variance, especially when they are trained on small datasets. On the other hand, RF with bootstrap aggregation and random feature selection gives strong variance reduction and robust generalization. By putting RF as a meta-learner on GBM outputs, the hybrid pipeline made the predictions of GBM stable, lowered the probability of overfitting, and improved the predictive performance of the system as a whole. This combination allowed simultaneous bias reduction (GBM) and variance control (RF), leading to a more reliable and well-calibrated prediction framework than the single models.

In the second stage of the study, ML techniques were employed to estimate the theoretical biological activity of a set of pyrazole derivatives. To reduce the dimensionality of the dataset and focus on the most informative molecular descriptors, feature selection was first carried out using RFE. Initially, a robust set of 273 molecular descriptors was calculated, which included the topological, physicochemical, geometric, and quantum-chemical properties. The next step was to apply RFE with cross-validation to shrink the dimensionality and get rid of the redundant features. RFE worked by removing the least informative descriptors iteratively based on the model’s performance, which at the end yielded a final optimized subset of 30 descriptors. This process was performed to strengthen predictive stability, eliminate multicollinearity, and reduce the risk of overfitting in the small dataset. In some cases, k-fold cross-validation was also utilized to better assess model stability and generalizability. To address potential overfitting, the models were evaluated using repeated 5-fold cross-validation, and all analyses were performed with fixed random seeds to ensure reproducibility. The tree-based algorithms (GBM and RF) inherently regularize model complexity through depth limitations, node splitting thresholds, and ensemble averaging. These steps were implemented to mitigate overfitting risks associated with the relatively small dataset. Before model training, all continuous descriptors were standardized using the StandardScaler (scikit-learn), applying Z-score normalization (scaler.fit_transform(X)). This preprocessing step ensured that features had comparable scales and prevented magnitude-based bias, particularly for algorithms sensitive to feature distributions. A range of algorithms were tested, including LR, DT, RF, GBM, and ANN. In addition to these individual models, hybrid approaches were explored to leverage complementary strengths. Notably, the RF+ANN combination and the custom-designed GBM+RF hybrid model delivered promising results. Model evaluation was based not only on standard performance metrics such as R^2^, mean absolute error (MAE), and root mean squared error (RMSE), but also on the Q^2^ statistic to ensure predictive reliability on unseen data. Overall, the proposed GBM+RF hybrid model stood out for its accuracy and robustness. All the ML models were initially implemented using their default hyperparameter settings, as provided by the scikit-learn library, prior to systematic hyperparameter optimization. The GBM model used a learning rate of 0.1, 100 boosting stages (n_estimators), 3 as maximum tree depth, a subsample ratio of 1.0, and squared-error loss function. The other model used for RF was composed of 100 DT, the criterion of squared-error, unlimited tree depth (max_depth = none), minimum split size of 2, minimum leaf size of 1, allowed bootstrap sampling, and the sqrt feature sampling strategy. The DT model was based on the squared-error criterion, max_depth = none, min_samples_split = 2, and min_samples_leaf = 1, and all feature selection settings were default. LR implemented L2 regularization with C = 1.0, the lbfgs solver, and 100 iterations limit. The ANN (MLPRegressor) had one hidden layer with 100 neurons, ReLU activation, the Adam optimizer, fixed learning rate of 0.001, and a maximum of 200 training iterations.

## 3. Results and discussion

The present study aimed to investigate the SAR of 54 pyrazole derivatives using an integrative framework that combined 4D-QSAR modeling with ML techniques. The findings demonstrated that the 4D-QSAR model successfully captured the conformational and electronic features governing IC_50_ variations across the series, while the ML models provided complementary predictive strength, yielding high accuracy and robustness. Rather than merely summarizing predictive metrics, the present study interpreted these modeling results in a broader biological and methodological context, suggesting that the selected descriptors and pharmacophoric features capture key structural determinants governing the activity of pyrazole derivatives. Together, these results highlight the importance of incorporating conformational flexibility and nonlinear descriptor–activity relationships in the modeling of heterocyclic scaffolds such as pyrazoles. The true value of the model therefore lies not only in its statistical accuracy, but also in its ability to provide chemically meaningful insights that may guide future compound design and prioritization strategies. These observations are consistent with previous reports indicating that pyrazole-based compounds exhibit strong SAR sensitivity to substituent orientation and electronic distribution, and that hybrid computational approaches can enhance the reliability of activity prediction. (Tao et al., 2007; Hanachi et al., 2021; Maattallaoui et al., 2025). Our integrated results provide additional evidence supporting the hypothesis that combining ensemble-based QSAR descriptors with data-driven learning algorithms can offer a more comprehensive and generalizable understanding of bioactivity patterns.

Compared with traditional 2D- and 3D-QSAR approaches, the proposed hybrid framework introduces several methodological innovations that directly enhance predictive performance. First, the incorporation of 4D-QSAR conformational ensemble descriptors enables the model to capture multiple low-energy spatial arrangements for each pyrazole analog, overcoming the limitations of single-conformer or static alignment-based QSAR models. Second, ML algorithms, particularly RF and XGBoost, allow capturing of nonlinear and higher-order interactions among steric, electronic, hydrophobic, and conformational descriptors, which classical regression-based QSAR methods typically fail to identify. Third, the hybrid feature-selection strategy reduces redundancy in the descriptor set, minimizes noise, and improves generalization on external test compounds. These combined innovations explain why the hybrid model shows superior predictive accuracy and robustness when compared with conventional QSAR techniques, while also offering enhanced mechanistic interpretability for structure-guided optimization of pyrazole derivatives.

To improve the interpretability of the predictive model, the key molecular descriptors contributing to bioactivity prediction were analyzed. The most influential variables were from to steric, electronic, hydrophobic, and conformational descriptor classes, all calculated using Spartan 0.7. Steric descriptors (e.g., VdW volume, molecular refractivity) showed positive correlations with activity, indicating that bulkier substituents at the C-3 and C-5 positions of the pyrazole core enhanced complementarity with the biological target. Electronic descriptors, including the HOMO–LUMO gap and dipole moment, suggested that molecules with balanced electron-distribution profiles exhibited stronger predicted activity. Hydrophobic descriptors such as the calculated logarithm of the partition coefficient and topological polar surface area were among the strongest contributors, consistent with previous findings demonstrating that lipophilic pyrazole scaffolds show improved membrane permeability and target engagement. Conformational descriptors derived from 4D-QSAR alignment fields indicated that low-energy accessible conformers significantly influence activity prediction, highlighting the mechanistic importance of conformational adaptability in ligand recognition. These trends are in agreement with recent hybrid QSAR-ML studies on pyrazole derivatives, which similarly emphasize the role of steric and electronic distribution in activity modulation (Chalkha et al., 2022; Mohamed et al., 2022; Chen et al., 2023b). Overall, the descriptor interpretation confirms that the model provides both predictive accuracy and mechanistic insight, thereby enhancing its value for future structure-guided design.

In the present investigation, 54 biological data points, sourced from the study conducted by Tao et al. (2007), were utilized. The biological activity data of these 54 pyrazole derivatives are summarized in Table 1, which shows that the dataset spanned a broad range of pIC_50_ values, with highly active compounds clustered around substitutions at the C-3 and C-5 positions of the pyrazole core. This wide activity distribution provided a suitable basis for both 4D-QSAR modeling and ML-based prediction. Notably, as shown in Table 1, compound **11**, which belongs to the test set, exhibited a pronounced deviation between experimental and predicted activity values. This deviation suggests that the model may be extrapolating beyond its optimal applicability domain for this compound, likely due to structural features that are insufficiently represented in the training set. In particular, the substituent pattern of compound **11** may introduce steric or electronic effects that are not fully captured by the selected descriptors. Despite this compound-specific deviation, the predictive performance of the model for the remaining test set compounds remained relatively consistent. Additionally, activity values calculated in pIC_50_ units based on the obtained κ_j_ values for the pyrazole series (1–12 κ) are presented in Tables S4 and S5. Quantum-chemical semiempirical calculations at the PM3 level were executed using Spartan 0.7 for all pyrazole derivatives. Semiempirical CNDO calculations performed in Spartan 0.7 were used exclusively for generating quantum-chemical descriptors, which were then incorporated into the 4D-QSAR modeling workflow. Electronic, steric, hydrophobic, and conformational descriptors obtained from these calculations were subsequently used as input in the EC-GA 4D-QSAR model. ECMCs were constructed employing electronic structure calculation and conformational analysis data for each compound in the series. EMRE software, built on the Delphi program developed by Borland Software^6^ (available online), and developed by our research group, facilitated the generation of ECMCs.

**Figure 3:**
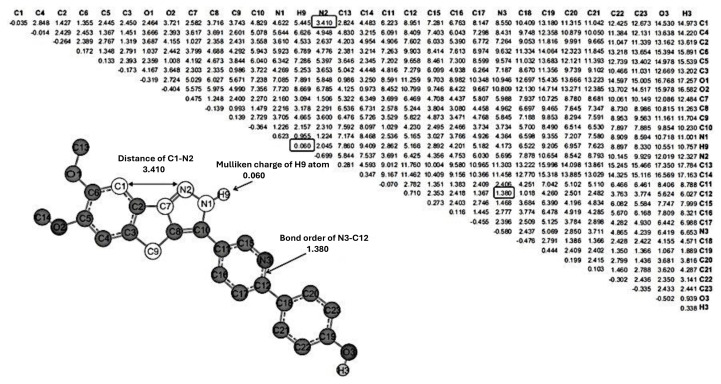
ECMC-derived lowest energy conformer of compound **27**. The figure shows the 2D representation of the molecule along with the ECMC matrix. Bond orders, Mulliken charges, and interatomic distances are indicated for key atoms, providing a detailed overview of the electronic and geometric properties of the molecule. This representation highlights the optimized geometry and electronic distribution obtained from the ECMC analysis.

Geometric and electronic values of the carbon–hydrogen bonds were excluded from consideration as they exhibit equal impacts in each compound (Figure 3). The ECMC of the lowest energy conformer with the highest activity, identified in compound **27**, was chosen as a reference pattern. This reference ECMC was then compared to the ECMCs of conformers with the lowest energy for other compounds, maintaining specified tolerances (Bersuker, 2003). In the EMRE program, during the process of determining the submatrix, unique numbers are assigned to Spartan 0.7 atoms for each compound, leading to potential confusion.

To overcome this challenge, an atom-type matrix (ATM) file was generated for each compound to correspond to the numbering of the atoms. This file was constructed based on the simplest structured compound. Figure S1 illustrates an example derived from the ATM file, referred to as such, which was generated by sequentially comparing and recording each atom in the pyrazole series compounds. As observed, the atoms in the fundamental skeleton of each compound in the series are superimposed and written in the file. The file significantly enhanced the speed of the program during atom comparisons, providing substantial convenience to our research group in the calculation of submatrices.

Through systematic adjustment of tolerance thresholds for nondiagonal components (such as bond orders or interatomic distances) and diagonal elements (charges), tolerance limits were iteratively derived, as presented in Table 2 of the ECSA, which demonstrates the optimal differentiation between active compounds and comparably small active or inactive compounds. In Table 2, the initial ECSA functions are given as the reference compounds, the second ECSA delineates tolerance values for highly active compounds, and the third submatrix exhibits tolerance values for less active compounds.

Following this, maximum tolerance values were calculated for all conformers of each compound without imposing restrictions on the pharmacophore’s tolerance values. The ultimate ECSA compiled tolerance values for all 54 compounds distributed among 1154 conformers. After inspecting the tolerances in the initial ECSA for 54 compounds, which was established with a maximum limit of 0.25 for diagonal elements and 1.30 for off-diagonal elements, the ECMC submatrix shared among all active molecules was found to include six atoms denoted as C1, C7, C9, H9, N1, and N2.

The unique characteristic of the EC technique lies in the adaptability of the Pha geometry and its consequential effects on the overall operation. It was observed that the tolerance matrix for compounds with higher activity did not align with the matrix for less active compounds, as illustrated in Table 2. In high-activity compounds, tolerance values were typically smaller compared to their counterparts with lower activity (Table 2). Two probabilistic estimates can be employed to assess the quality of a property (Dimoglo et al., 2001):


(12) $$P_{a}=\frac{h_{1}+1}{h_{1}+h_{2}+2},$$

(13) $$\alpha_{a}=\frac{h_{1}{\text{h}}_{4}-h_{2}h_{3}}{k_{1}k_{2}k_{3}k_{4}}.$$

Here, h_1_ and h_2_ represent the quantities of molecules containing or lacking activity characteristics, respectively, chosen by the ECM in the active compound class. Similarly, h_3_ and h_4_ denote the corresponding values for small activity molecules. The variables k_1_ and k_2_ represent the total number of active and low-activity molecules, respectively, while k_3_ is defined as h_1_ + h_3_, and k_4_ is defined as h_2_ + h_4_. These parameters elucidate the likelihood of property fulfillment being deemed favorable. Values greater than 0.6 are generally considered sufficient. The calculated values were P*_α_* = 0.8402 and *α_a_* = 0.8261.

To enhance the methodology and predict anticancer activity, 204 parameters encompassing electronic, topological, structural, and physicochemical constants were considered as potential input candidates in the model. Through the elimination–selection procedure implemented within the GA, a total of 12 parameters were selected. According to the rule of thumb, the size of the information set should be approximately five times larger than the number of selected parameters to ensure robust and reliable model performance. Beyond their statistical relevance, the importance of each selected parameter was further examined based on its underlying physicochemical meaning.

In this study, the GA codes were implemented in MATLAB and executed on parallel computing platforms with multiple processors and cores. In Eq. (2), the adjustable constants, κ_j_, were optimized using the *lsqnonlin* function available in the MATLAB optimization toolbox. As illustrated in Figure 3, the optimal subset of 12 parameters represents those descriptors that most effectively enhanced biological activity while reflecting the flexibility of ECSA-derived features.

**Figure 4:**
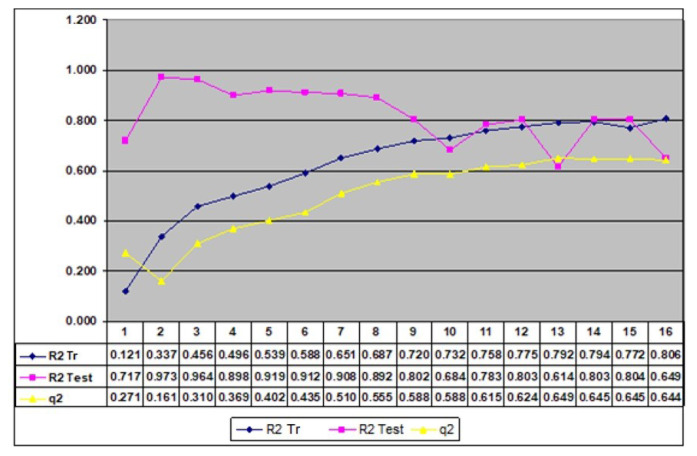
Regression coefficient plot for the pyrazole series with model validation metrics. The figure shows the regression coefficients for the optimal number of parameters, indicating the contribution and direction of each molecular descriptor to the predicted activity. Model performance metrics, including R^2^_training_, R^2^_test_, and *Q*^2^ , are provided to demonstrate the goodness-of-fit, predictive accuracy, and cross-validated reliability of the model.

Notably, these parameters encode chemically meaningful information related to electronic distribution, molecular flexibility, and nuclear groupings beyond the core pharmacophore. After the inclusion of these 12 descriptors, the model reached stability, and the incorporation of additional parameters did not yield further improvement, as shown in Figure 4. The final optimal parameter set, a(j), shown in Table 3, was therefore selected from extensive GA test calculations to capture the influence of ECSA parameter flexibility and extra-pharmacophoric nuclear groupings on biological activity.

The efficiency of the suggested EC-GA approach was clarified through diverse evaluation methods: R^2^, representing the square of the correlation coefficient; q^2^, indicating the cross-validated R^2^; validation utilizing an external test set, and s, denoting the standard error. The cross-validated R^2^, determined through the LOO method introduced by Cramer et al. (1988), offers insights into the method’s robustness. Earlier studies suggested that models with a q^2^ value exceeding 0.5 were considered to possess predictive capabilities superior to estimation, and higher q^2^ values were associated with increased credibility (Golbraikh and Tropsha, 2002). Consequently, the model underwent additional refinement to improve its q^2^ and, thereby, its predictive efficacy.

An additional statistical tool, the E statistic, of significant practical value, is presented in Table 4. Subsequently, employing the GA, the optimal subset of descriptors was chosen from the extended set, and E statistics were computed as a metric to evaluate how accurately the estimation of bioactivity was influenced by each parameter. The following formulas were utilized (Makkouk et al., 2004):


(14) $$E=\frac{PRESS_{N}}{PRESS_{N-1}},$$

in which


(15) $$PRESS_{N-1}=\Sigma_{n=1}^{N-1}|A_{n}^{exp}-A_{n}^{calc}{|}^{2}.$$

Here, A_n_^calc^ represents the predicted and A_n_^exp^ is the experimental activities in the LOO-CV model, with N parameters being utilized (in this instance, N = 12).

The descriptors in Table 4 reveal that a ^(1)^, a ^(5)^, a ^(8)^, a ^(11)^, and a ^(12)^ possessed the lowest E values, establishing them as the most influential descriptors. This observation was corroborated by the substantial decrease in the R^2^ value, dropping from 0.775 to 0.676, 0.718, 0.669, 0.698, and 0.699, respectively. Notably, excluding a ^(6)^ and a ^(9)^ resulted in a negligible loss of precision, with q^2^ decreasing only from 0.624 to 0.610. In summary, the effectiveness of these descriptors aligned with the outcomes derived from E statistics, and it is evident that the GA is well-aligned with these results.

Model commentary plays a crucial role in QSAR, as it enhances the ability to regulate molecules. Without it, the regulatory capacity is limited. Nonlinear models, often considered challenging to interpret, are sometimes referred to as blackbox models (Fox and Kriegl, 2006). In the context of discovering potentially active compounds using the EC-GA method, structural data about the new compound become imperative. In the EC-GA method, the process involves quantum-chemical calculations to obtain geometric, electronic, and thermodynamic properties of the new molecule’s structure. Subsequently, bioactivity is estimated using selected parameters and their κ coefficients in Eq. (1) for the respective molecule. This approach ensures a comprehensive understanding of the molecular properties and their correlation with bioactivity, enabling the identification of novel, potentially active compounds. We acknowledge that the EC-GA pharmacophore modeling approach carries inherent limitations. The methodology assumes rigid alignment of ligand features, additive contributions of pharmacophoric elements, and does not explicitly account for receptor flexibility, solvation effects, or entropic contributions. Additionally, the predictive performance of the model is dependent on the size and chemical diversity of the ligand dataset, which may limit its generalizability. Future studies could address these limitations by expanding the ligand set, incorporating receptor-based flexibility, and considering dynamic and environmental effects to improve model reliability.

**Figure 5:**
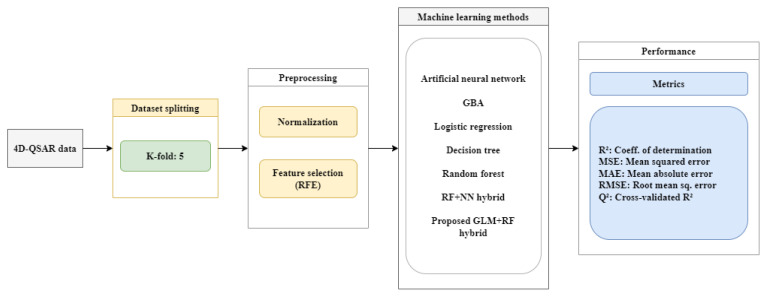
Workflow of the ML experiments for the 4D-QSAR dataset. The diagram illustrates the complete experimental pipeline, starting with dataset preparation and K-fold (k = 5) splitting. Data preprocessing steps include normalization and feature selection using RFE. Multiple ML methods were applied, including ANN, LR, DT, RF, hybrid RF+NN hybrid, and the proposed GLM+RF hybrid model. Model performance was evaluated using metrics such as *R*^2^, MSE, MAE, RMSE, and *Q*^2^, providing a comprehensive assessment of predictive accuracy and reliability.

In this study, various ML algorithms were used to compare the performance of different predictive models. The methods evaluated include ANN, GB, LR, DT, and RF. These techniques were assessed both individually and, in some cases, as part of hybrid combinations. The predictive performance of each model was analyzed using statistical metrics such as R^2^, MSE, MAE, RMSE, and Q^2^ for both the training and test sets. Figure 4 represents the distribution of prediction errors for both the training and test sets, highlighting the overall stability of the models while also revealing compound-specific deviations. The main objective of the study was to identify the best-performing model with the lowest error rate and the highest explanatory power. Table 5 shows the various ML algorithms that were employed to evaluate and compare the performance of different predictive models. In the experiments, 5-fold cross-validation was used to evaluate the model more reliably. This setup provided a better understanding of how well the model performs on different parts of the data and made the results more trustworthy. As a result of the analysis using the RFE method, 30 selected features were identified (Table 5). These features represented the baseline configurations employed prior to hyperparameter tuning and served as the starting point for the evaluation of model performance. Figure 5 shows the flowchart of the experiments performed with ML.

**Figure 6:**
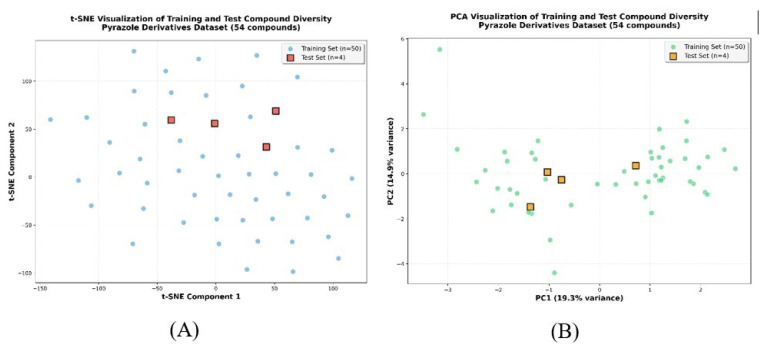
t-SNE and PCA visualization of chemical space coverage for the training and test compounds. (A) t-SNE plot showing the distribution of training (n = 50, blue circles) and test (n = 4, red squares) compounds in 2D space. (B) PCA plot displaying the same dataset with explained variance ratios for PC1 (19.3%) and PC2 (14.9%). Both visualizations demonstrate that the test set compounds are well-distributed across the chemical space occupied by the training set, ensuring adequate representation for model validation.

To evaluate the representativeness of the data split, t-distributed stochastic neighbor embedding (t-SNE) and PCA (Giuliani, 2017) dimensionality reduction analyses were performed on the molecular descriptor space (Figure 6). Both visualizations demonstrated that the four test compounds were well-distributed across the chemical space occupied by the 50 training compounds, rather than being confined to a single region. This distribution pattern is essential for unbiased model validation, as it ensures that the test set predictions reflect true generalization capability across diverse pyrazole structures. The PCA showed that PC1 and PC2 captured 19.3% and 14.9% of the variance, respectively, while the t-SNE plot revealed the nonlinear relationships among the compounds. This spatial analysis validated the appropriateness of the training-test split for robust model evaluation.

In Table 6, the proposed hybrid ML model combining GBM and RF demonstrated outstanding predictive performance in estimating the biological activity of pyrazole derivatives. The model successfully leveraged the sequential error correction capability of GBM and the variance-reducing ensemble structure of RF. When applied to the same dataset, the GBM+RF hybrid model achieved an R^2^ value of 0.99978 and a cross-validated predictive squared correlation coefficient (Q^2^) of 0.99928, indicating both exceptional accuracy and robust generalizability. This hybrid framework outperformed the individual models and other hybrid configurations tested in this study, particularly in handling nonlinearities and minimizing overfitting. Therefore, the GBM+RF hybrid model is proposed as a problem-specific, effective, and generalizable prediction strategy for modeling complex SAR in anticancer drug discovery.

The new models that we have suggested remain very strong, however, we accept that the extremely high R^2^ values (0.999) should be treated cautiously since the dataset is small. Small sample sizes commonly increase the risks of overfitting where a model memorizes training and does not learn the underlying generalizable SAR. To reduce this risk, various measures were taken. First, the single train–test split method was not used to assess model performance; rather, repeated 5-fold cross-validation was conducted to achieve a more dependable estimation of generalization. Second, the original descriptor space of 273 variables was narrowed down to 30 via RFE, hence reducing the complexity of the model and eliminating the chance of fitting by noise. Moreover, the overfitting of ensemble tree models was decreased by the regularization mechanisms they possess, such as controlling the depth, learning rate, and bootstrap aggregation. The alignment that was observed between cross-validated predictions and the independent external test set supported the assertion that the model was uncovering significant physicochemical patterns rather than relying on memorization. Nonetheless, we acknowledge that the size of the dataset is a major drawback, and future research will focus on increasing the compound set size to enable more solid external validation (Schüürmann et al., 2008) and improved generalization performance.

**Figure 7:**
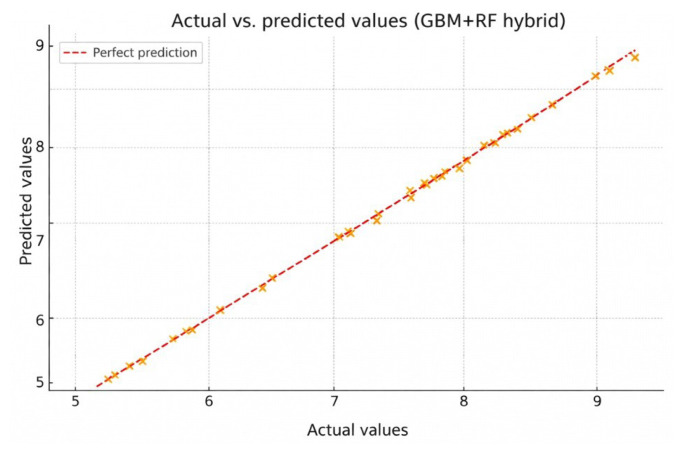
Comparison of actual versus predicted biological activity values for the pyrazole series using the GBM+RF hybrid model. The scatter plot illustrates the predictive performance of the model, where the x-axis represents experimentally measured activities and the y-axis shows the corresponding predicted values. The solid line indicates the ideal fit (y = x), highlighting the agreement between observed and predicted activities.

Figure 7 compares the biological activity values predicted by the GBM+RF hybrid model with the actual observed values. Strong agreement between the predictions and real values is clearly visible, as the data points are densely clustered around the ideal linear line. This indicates that the model operates with high accuracy and possesses strong generalization capability. The plot further confirms that the model effectively learns complex structural features and provides reliable results in predicting biological activity.

In summary, the main objective of this study was to establish a distinctive relationship between structural characteristics and inhibitory activity, employing an improved EC-GA 4D-QSAR method that integrates EC and GA techniques. The study focused on 54 chemically diverse molecules within the substituted pyrazole class. The primary goal of the quantitative model was to identify active structures and accurately predict their anticancer activity. To validate the model’s predictive capabilities for structurally distinct compounds, a test set of four molecules with varying activity classes and structural knowledge was utilized. The construction of the pharmacophore model for pyrazole derivatives involved the use of the EMRE software package, resulting in several models. At every stage of the EC-GA modeling, multiconformation for the compounds was considered based on Boltzmann weights. The structures in the dataset were aligned with a reference compound in a specified conformation, adhering to the lowest tolerance values. As the alignment relied on the relative positions of shared atoms in space, the 4D-QSAR technique employed in this study was geometric in nature. The optimal pharmacophore model identified six atoms: C1, C7, C9, H9, N1, and N2. The pharmacophore model can also be utilized for identifying analogous action mechanisms within molecular databases. The relevance of these pharmacophoric features is supported by previous SAR studies on pyrazole derivatives, which emphasize the critical role of substituent positioning and electronic distribution around the pyrazole core in modulating biological activity. In particular, substitutions at carbon and nitrogen positions of the pyrazole ring have been shown to influence target binding through steric complementarity and hydrogen-bonding interactions, consistent with the involvement of C1, C7, N1, and N2 identified in the present model. The pharmacophoric features identified in this study are consistent with known SAR trends of pyrazole derivatives reported in the literature. Pyrazole scaffolds are well-recognized as versatile pharmacophores, and structural modifications at positions such as C-1, C-3, and C-5 have been shown to significantly influence biological activity across diverse targets, including antiinflammatory, anticancer, and antimicrobial profiles, due to electronic and steric effects of the substituents (e.g., celecoxib, rimonabant, and other pyrazole-based drugs), which underscore the importance of functional group orientation on activity binding (Karrouchi et al., 2018). Specifically, electron-donating or withdrawing groups at these positions and heteroaromatic substituents have been reported to modulate potency and selectivity by altering hydrophobic interactions and hydrogen bonding within target binding sites, supporting the chemical relevance of the modeled pharmacophore features such as C1, C7, and N1 (Li et al., 2022). These consistencies suggest that our pharmacophore model captures known SAR motifs rather than reflecting purely statistical correlations.

In addition to the EC-GA 4D-QSAR approach, a hybrid ML model combining GBM and RF was also proposed to enhance predictive performance. This model effectively integrated the sequential learning strength of GBM with the ensemble variance-reduction capability of RF. When applied to the same dataset, the hybrid model achieved exceptionally high R^2^ and Q^2^ values, indicating its superior predictive accuracy and robustness in modeling QSAR.

Furthermore, the GA and least square minimization technique were applied, relying on the Pha, to determine the most significant parameters affecting the activity and their corresponding κj values in predicting theoretical activity values. Higher GA parameters (population = 500, generations = 500, iterations = 500) were selected after preliminary optimization. Lower settings (50–200), which are adequate for simpler datasets, resulted in premature convergence and unstable fitness values in this multidescriptor, multiconformer 4D-QSAR system. Therefore, increasing the GA search depth was necessary to obtain stable convergence and improved predictive performance. Subsequently, 204 molecular descriptors for each compound were computed using Spartan 0.7 and collected via EMRE software. To reduce descriptor redundancy and prevent overfitting, a GA-based feature selection strategy was applied, and only statistically significant descriptors with low intercorrelation were retained for model construction. For 4D-QSAR modeling, a training set of 50 compounds was utilized, while the remaining four molecules served as an internal test subset to evaluate the models. The findings indicated that anticancer activity could be effectively modeled using physicochemical constants and structural parameters. The validation techniques employed in the research not only assessed the model’s fitness on the training set but also evaluated its predictive capacity on unseen compounds. With its elevated predictive accuracy, the proposed approach, supported by the integration of EC-GA-based 4D-QSAR and ML hybrid modeling, offers a practical and computationally efficient alternative to costly and time-consuming experimental studies in the identification of pyrazole anticancer activity. Accordingly, the hybrid QSAR-ML framework presented herein may be effectively applied to virtual screening, lead optimization, and structure-guided design of pyrazole-based anticancer candidates.

The predictive performance achieved in this study is comparable to, and in some respects, complementary with, previously reported QSAR and ML models developed for pyrazole derivatives and related heterocyclic scaffolds. Earlier studies primarily relied on classical 2D/3D-QSAR or single ML algorithms, often focusing on linear descriptor–activity relationships and fixed conformations. In contrast, the present work integrates 4D-QSAR-based conformational sampling with nonlinear ML models, enabling the capture of both conformational flexibility and complex interactions among descriptors. While direct numerical comparison across studies is limited by differences in datasets and activity endpoints, the observed performance metrics and mechanistic interpretability suggest that the proposed hybrid framework offers a robust and chemically meaningful alternative to existing QSAR/ML approaches for pyrazole-based compounds.

Despite the strengths of the 4D-QSAR hybrid and ML framework, several limitations should be acknowledged. First, the dataset consisted of 54 pyrazole derivatives obtained from a single literature source, which may limit chemical diversity and reduce the generalizability of the model beyond this scaffold. Second, quantum-chemical descriptors were generated at the semiempirical PM3 level using Spartan 0.7, which, although computationally efficient, may introduce approximations compared with higher-level ab initio or DFT methods. Third, conformational ensemble sampling was constrained by the capabilities of Spartan 0.7, and therefore some high-energy or transient conformers may not have been fully explored. Fourth, although ML algorithms improved predictive accuracy, validation relied on internal cross-validation and an external test split, and no prospective or experimental validation was performed. Finally, the absence of explicit receptor-based simulations, such as molecular docking or molecular dynamics, limited direct mechanistic interpretation of binding interactions. These limitations highlight opportunities for future studies involving larger and more diverse datasets, higher-level quantum calculations, extended conformational sampling, and integration with receptor-based modeling or experimental validation.

## Supplementary Information



## Figures and Tables

**Table 1:** Experimental (A^exp^) and predicted (A^calc^) pIC_50_ values for the studied compounds, summarized and classified into high and low activity groups based on experimental pIC_50_ values. Predicted activities were obtained using the optimal EC-GA-based ML model. The complete ungrouped numerical dataset for all 54 compounds is provided in Table S5.

Compound ID	Experimental pIC_50_ (A^exp^)	Predicted pIC_50_ (A^calc^)	Activity class
**1**	8.699	8.255	High-activity
**2**	7.602	7.871	Low-activity
**3**	8.155	7.769	High-activity
**4**	8.699	8.784	High-activity
**5**	7.602	7.886	Low-activity
**6**	6.108	5.638	Low-activity
**7**	6.450	6.125	Low-activity
**8**	5.000	5.342	Low-activity
**9**	7.137	6.108	Low-activity
**10**	5.000	6.704	Low-activity
**11**	5.890	3.370	Low-activity
**12**	5.206	5.370	Low-activity
**13**	5.339	4.533	Low-activity
**14**	7.959	7.643	High-activity
**15**	7.328	7.825	Low-activity
**16**	7.114	7.258	Low-activity
**17**	5.900	6.050	Low-activity
**18**	5.862	5.163	Low-activity
**19**	5.431	6.451	Low-activity
**20**	5.522	6.439	Low-activity
**21**	5.289	5.481	Low-activity
**22**	7.721	6.644	High-activity
**23**	8.301	7.494	High-activity
**24**	5.000	5.355	Low-activity
**25**	5.759	5.940	Low-activity
**26**	5.000	4.957	Low-activity
**27**	9.301	9.301	High-activity
**28**	9.097	8.755	High-activity
**29**	7.824	7.630	High-activity
**30**	8.398	7.526	High-activity
**31**	8.699	7.277	High-activity
**32**	6.541	7.866	Low-activity
**33**	6.541	6.917	Low-activity
**34**	7.796	7.635	High-activity
**35**	5.000	4.572	Low-activity
**36**	7.357	7.323	Low-activity
**37**	7.051	6.647	Low-activity
**38**	7.357	7.368	Low-activity
**39**	8.398	8.331	High-activity
**40**	8.523	8.252	High-activity
**41**	8.523	8.962	High-activity
**42**	8.222	8.654	High-activity
**43**	7.854	8.311	High-activity
**44**	7.699	9.410	High-activity
**45**	8.000	7.422	High-activity
**46**	7.114	7.299	Low-activity
**47**	8.046	6.612	High-activity
**48**	8.699	8.915	High-activity
**49**	8.523	8.462	High-activity
**50**	8.699	8.342	High-activity
**51**	9.000	7.799	High-activity
**52**	8.523	8.293	High-activity
**53**	7.770	8.583	High-activity
**54**	8.699	9.277	High-activity

**Table 2:** (a) ECSA values for the reference compound (**27**) in pyrazole derivatives. (b) Tolerance matrix of ECSA values for 28 compounds exhibiting high activity. (c) Tolerance values for 26 compounds with low activity. (d) Tolerance values for 1154 conformations of the 54 compounds.

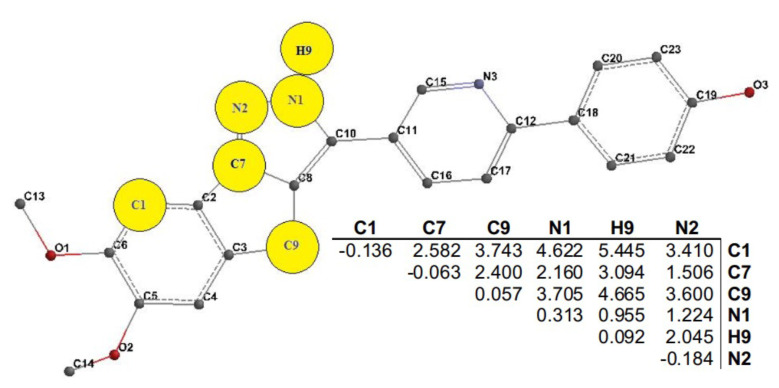					
C1	C7	C9	N1	H9	N2
**(a) ECSA (Pha) of reference compound**					
−0.136	2.582	3.743	4.622	5.445	3.410
	−0.063	2.400	2.160	3.094	1.506
		0.057	3.705	4.665	3.600
			0.313	0.955	1.224
				0.092	2.045
					−0.184
**(b) Tolerance values for 28 compounds exhibiting significant activity.**					
±0.094	±0.003	±0.005	±0.006	±0.008	±0.009
	±0.017	±0.003	±0.003	±0.003	±0.004
		±0.008	±0.006	±0.005	±0.003
			±0.008	±0.002	±0.010
				±0.001	±0.003
					±0.010
**(c) Tolerance values for 26 compounds with low activity compounds**					
±0.090	±0.003	±0.002	±0.008	±0.009	±0.007
	±0.014	±0.003	±0.007	±0.003	±0.028
		±0.013	±0.009	±0.004	±0.003
			±0.054	±0.002	±0.037
				±0.010	±0.006
					±0.013
**(d) Tolerance values for 1154 conformations across 54 compounds.**					
±0.094	±0.005	±0.007	±0.009	±0.012	±0.012
	±0.017	±0.003	±0.004	±0.003	±0.029
		±0.021	±0.009	±0.006	±0.003
			±0.054	±0.006	±0.038
				±0.010	±0.006
					±0.018

**Table 3:** Optimal set of 12 molecular parameters selected using the GA, along with their corresponding κj values, which were incorporated into the activity calculation and collectively define the contribution of each descriptor to the final predictive model.

a^(j)^	Parameter Number	Molecular Parameters	K_j_ values
**a ^(1)^**	13	Mulliken Charge of N2 Atom	−17.633
**a ^(2)^**	17	Mulliken Charge of C13 Atom	10.552
**a ^(3)^**	19	Mulliken Charge of O3 Atom	0.354
**a ^(4)^**	59	Polarizability ZYY	−0.001
**a ^(5)^**	84	The perpendicular distance of the O3 atom to the C1-H9-C9 plane	1.130
**a ^(6)^**	96	The angle between the C1-H9-C9 plane and the O3-C11 line	0.172
**a ^(7)^**	101	The angle between the C1-H9-C9 surface and the O3-C10 line	−0.347
**a ^(8)^**	121	The angle between H9-N1-N2	1.274
**a ^(9)^**	149	The logarithm of the distance between O3 and N1	−0.327
**a ^(10)^**	199	PSA (Polar Surface Area)	−0.004
**a ^(11)^**	202	Rel E(aq)	0.001
**a ^(12)^**	204	The torsion angle of O3-O2-C5-C7	0.001

**Table 4:** E, R^2^_Training_, R^2^_Test_ q^2^, q^2^_ext1_, and q^2^_ext2_ statistical metrics showing the individual contribution of each selected parameter to model fitting, internal validation, and external predictive performance, thereby providing a comprehensive assessment of model robustness and generalization capability.

*Eliminated Parameters*		E	R^2^_Training_	R^2^_Test_	q^2^	q_2ext_1	q^2^_ext2_
***a *** *^(1)^*	13	0.721	0.676	0.860	0.478	−3.878	−3.885
***a *** *^(2)^*	17	0.863	0.737	0.830	0.564	−2.533	−2.539
***a *** *^(3)^*	19	0.909	0.746	0.800	0.586	−3.871	−3.878
***a *** *^(4)^*	59	0.892	0.732	0.911	0.578	−0.659	−0.661
***a *** *^(5)^*	84	0.711	0.718	0.787	0.471	−2.760	−2.766
***a *** *^(6)^*	96	0.965	0.758	0.802	0.610	−3.210	−3.216
***a *** *^(7)^*	101	0.907	0.751	0.801	0.585	−3.221	−3.227
***a *** *^(8)^*	121	0.624	0.669	0.712	0.398	−7.065	−7.077
***a *** *^(9)^*	149	0.964	0.756	0.812	0.610	−2.043	−2.048
***a *** *^(10)^*	199	0.847	0.736	0.788	0.556	−2.238	−2.243
***a *** *^(11)^*	202	0.756	0.698	0.829	0.502	−2.147	−2.152
***a *** *^(12)^*	204	0.665	0.699	0.930	0.434	−4.067	−4.075

**Table 5:** Features selected using RFE to identify the most informative descriptors contributing to model performance.

Selected Features	Selected Features
**i_desc_Chirality_count**	**r_desc_Mean_Distance_Degree_Deviation**
**i_desc_Polarity**	**r_desc_Molecular_electrotopological_variation**
**i_desc_Ring_Count_5**	**r_desc_PEOE1**
**i_desc_Ring_Count_6**	**r_desc_Petitjean_2D_shape**
**i_desc_Second_Zagreb**	**r_desc_Radial_centric**
**i_desc_Sum_of_topological_distances_between_N..Br**	**r_desc_Solvation_connectivity_index_chi-5**
**i_desc_Sum_of_topological_distances_between_N..F**	**r_desc_Topological_charge_index_of_order_8**
**i_desc_Sum_of_topological_distances_between_O..F**	**r_desc_Valence_connectivity_index_chi-5**
**i_desc_Topological_radius**	**r_desc_reciprocal_distance_square_Randic-type_index**
**r_desc_ALOGP3**	**i_qp_#NandO**
**r_desc_ALOGP7**	**i_qp_#acid**
**r_desc_Connectivity_index_chi-4**	**i_qp_#stars**
**r_desc_E-state_topological_parameter**	**i_qp_HumanOralAbsorption**
**r_qp_SAfluorine**	**r_qp_QPlogPo/w**
**r_qp_accptHB**	**r_qp_donorHB**

**Table 6:** The various ML algorithms employed to compare the performance of different predictive models, enabling an objective evaluation of their predictive accuracy, robustness, and generalization based on multiple statistical metrics.

Model	R^2^	MSE	MAE	RMSE	Q^2^
**ANN**	0.97155	0.00101	0.02207	0.03174	0.97892
**GB**	0.97757	0.00418	0.05274	0.06469	0.97707
**LR**	0.98280	0.02964	0.14936	0.17217	0.98230
**DT**	0.89457	0.18168	0.39645	0.42624	0.89407
**RF**	0.87526	0.21496	0.37864	0.46363	0.87476
**RF+NN Hybrid**	0.81275	0.32269	0.50284	0.56806	0.81225
**GBM+RF Hybrid**	0.99978	0.00038	0.01077	0.01962	0.99928

## Footnotes

1 National Science Foundation (2019). National Patterns of R&D Resources [online]. Website: https://ncses.nsf.gov/pubs/nsf22320/ [accessed 21 January 2026].

2 Wavefunction Inc. (2006). SPARTAN’07 for Windows and Linux [online]. Website: https://www.wavefun.com [accessed 21 January 2026].

3 The MathWorks Inc. (2012). MATLAB and Statistics Toolbox [online]. Website: https://www.mathworks.com/ [accessed 21 January 2026].

4 Tripos Inc. (2000). SYBYL Theory Manual, Version 6.7 [online]. Website: https://www.tripos.com [accessed 21 January 2026].

5 Schrödinger LLC (2023). Molecular Descriptors Module. Schrödinger Release 2023 2 [online]. Website: https://www.schrodinger.com [accessed 21 January 2026].

6 Borland Software (n.d.). Delphi programming environment [online]. Website: http://www.borland.com/delphi [accessed 21 January 2026].

## References

[b1-tjb-50-01-37] Ahmed AHH Mohamed MFA Beshr EMA 2024 A mini review on some biological activities of pyrazole derivatives International Journal of Pharmaceutical Sciences and Drug Analysis 4 2 34 40 10.22271/27889246.2024.v4.i2a.99

[b2-tjb-50-01-37] Altun A Golcuk K Kumru M Jalbout AF 2003 Electron-conformational study for the structure-hallucinogenic activity relationships of phenylalkylamines Bioorganic & Medicinal Chemistry 11 18 3861 3868 10.1016/S0968-0896(03)00437-1 12927846

[b3-tjb-50-01-37] Andrade CH Pasqualoto KFM Ferreira EI Hopfinger AJ 2010 4D-QSAR: Perspectives in Drug Design Molecules 15 5 3281 3294 10.3390/molecules15053281 20657478 PMC6263259

[b4-tjb-50-01-37] Becker OM Levy Y Ravitz O 2000 Flexibility, conformation spaces, and bioactivity The Journal of Physical Chemistry B 104 9 2123 2135 10.1021/jp992268m

[b5-tjb-50-01-37] Bernazzani L Duce C Micheli A Mollica V Sperduti A 2006 Predicting physical-chemical properties of compounds from molecular structures by recursive neural networks Journal of Chemical Information and Modeling 46 5 2030 2042 10.1021/ci060104e 16995734

[b6-tjb-50-01-37] Bersuker IB 2003 Pharmacophore identification and quantitative bioactivity prediction using the electron-conformational method Current Pharmaceutical Design 9 20 1575 1606 10.2174/1381612033454586 12871060

[b7-tjb-50-01-37] Bersuker IB 2008 QSAR without arbitrary descriptors: the electron-conformational method Journal of Computer-Aided Molecular Design 22 6–7 423 430 10.1007/s10822-008-9191-x 18283420

[b8-tjb-50-01-37] Bersuker IB Bahceci S Boggs JE Pearlman RS 1999a A novel electron-conformational approach to molecular modeling for QSAR by identification of pharmacophore and anti-pharmacophore shielding SAR and QSAR in Environmental Research 10 2–3 157 173 10.1080/10629369908039174 22091549

[b9-tjb-50-01-37] Bersuker IB Bahceci S Boggs JE Pearlman RS 1999b An electron-conformational method of identification of pharmacophore and anti-pharmacophore shielding: application to rice blast activity Journal of Computer-Aided Molecular Design 13 4 419 434 10.1023/A:1008052914704 10425606

[b10-tjb-50-01-37] Breiman L 2001 Random forests Machine Learning 45 5 32 10.1023/A:1010933404324

[b11-tjb-50-01-37] Chalkha M Akhazzane M Moussaid FZ Daoui O Nakkabi A 2022 Design, synthesis, molecular docking, 3D-QSAR and ADME-Tox of pyrazole derivatives New Journal of Chemistry 46 2747 2760 10.1039/D1NJ05621B

[b12-tjb-50-01-37] Chen L Lin Y Yan X Ni H Chen F 2023a 3D-QSAR studies on the structure–bitterness analysis of citrus flavonoids Food & Function 14 10 4921 4930 10.1039/D3FO00601H 37158134

[b13-tjb-50-01-37] Chen XD Li J Wang X Liu R Liu X 2023b 3D-QSAR, docking and MD of pyrazole derivatives as MALT1 inhibitors New Journal of Chemistry 47 19596 19607 10.1039/D3NJ03490A

[b14-tjb-50-01-37] Consonni V Ballabio D Todeschini R 2009 Comments on the definition of the Q^2^ parameter for QSAR validation Journal of Chemical Information and Modeling 49 7 1669 1678 10.1021/ci900115y 19527034

[b15-tjb-50-01-37] Cramer RD Patterson DE Bunce JD 1988 Comparative molecular field analysis (CoMFA). 1. Effect of shape on binding of steroids to carrier proteins Journal of the American Chemical Society 110 18 5959 5967 10.1021/ja00226a005 22148765

[b16-tjb-50-01-37] Devi RV Siva Sathya S Coumar MS 2021 Multi-objective genetic algorithm for de novo drug design (MoGADdrug) Current Computer-Aided Drug Design 17 3 445 457 10.2174/1573409916666200620194143 32562528

[b17-tjb-50-01-37] Dimoglo AS Vlad PF Shvets NM Coltsa MN Güzel Y 1995 Electronic-topological investigations of the relationship between chemical structure and ambergris odor New Journal of Chemistry 19 12 1217 1226

[b18-tjb-50-01-37] Dimoglo AS Shvets NM Tetko IV Livingstone DJ 2001 Electronic-topological investigation of the structure-acetylcholinesterase inhibitor activity relationship in the series of N-benzylpiperidine derivatives Quantitative Structure-Activity Relationships 20 1 31 45 10.1002/1521-3838(200105)20:1<31::AID-QSAR31>3.0.CO;2-S

[b19-tjb-50-01-37] Duca JS Hopfinger AJ 2001 Estimation of molecular similarity based on 4D-QSAR analysis: formalism and validation Journal of Chemical Information and Computer Sciences 41 5 1367 1387 10.1021/ci0100090 11604039

[b20-tjb-50-01-37] Fernandez M Caballero J Fernandez L Sarai A 2011 Genetic algorithm optimization in drug design QSAR: Bayesian-regularized genetic neural networks (BRGNN) and genetic algorithm-optimized support vector machines (GA-SVM) Molecular Diversity 15 1 269 289 10.1007/s11030-010-9234-9 20306130

[b21-tjb-50-01-37] Fox T Kriegl JM 2006 Machine learning techniques for in silico modeling of drug metabolism Current Topics in Medicinal Chemistry 6 15 1579 1591 10.2174/156802606778108915 16918470

[b22-tjb-50-01-37] Friedman JH 2001 Greedy function approximation: A gradient boosting machine Annals of Statistics 29 5 1189 1232 10.1214/aos/1013203451

[b23-tjb-50-01-37] Gillet VJ Khatib W Willett P Fleming PJ Green DVS 2002 Combinatorial library design using a multiobjective genetic algorithm Journal of Chemical Information and Computer Sciences 42 2 375 385 10.1021/ci010375j 11911707

[b24-tjb-50-01-37] Giuliani A 2017 The application of principal component analysis to drug discovery and biomedical data Drug Discovery Today 22 7 1069 1076 10.1016/j.drudis.2017.01.005 28111329

[b25-tjb-50-01-37] Golbraikh A Tropsha A 2002 Beware of q^2^! Journal of Molecular Graphics and Modeling 20 4 269 276 10.1016/S1093-3263(01)00123-1 11858635

[b26-tjb-50-01-37] Goldstein H Poole CP Safko JL 2001 Classical Mechanics 3rd ed Pearson

[b27-tjb-50-01-37] Gramatica P Pilutti P Papa E 2004 Validated QSAR prediction of OH tropospheric degradability of volatile organic compounds: Splitting into training–test sets and consensus modeling Journal of Chemical Information and Computer Sciences 44 5 1794 1802 10.1021/ci049923u 15446838

[b28-tjb-50-01-37] Guha R Jurs PC 2004 Development of linear, ensemble, and nonlinear models for the prediction and interpretation of the biological activity of a set of PDGFR inhibitors Journal of Chemical Information and Computer Sciences 44 6 2179 2189 10.1021/ci049849f 15554688

[b29-tjb-50-01-37] Hanachi R Ben Said R Allal H Rahali S Alkhalifah MAM 2021 Structural, QSAR, machine learning and molecular docking studies of 5-thiophen-2-yl pyrazole derivatives as potent and selective cannabinoid-1 receptor antagonists New Journal of Chemistry 45 17796 17807 10.1039/D1NJ02261J

[b30-tjb-50-01-37] Hehre WJ 2003 Molecular Mechanics & Quantum Calculations Wavefunction Inc

[b31-tjb-50-01-37] Hervás C Silva M Serrano JM Orejuela E 2004 Heuristic extraction of rules in pruned artificial neural networks models used for quantifying highly overlapping chromatographic peaks Journal of Chemical Information and Computer Sciences 44 5 1576 1584 10.1021/ci049948t 15446815

[b32-tjb-50-01-37] Hong X Hopfinger AJ 2003 3D-pharmacophores of flavonoid binding at the benzodiazepine GABA(A) receptor site using 4D-QSAR analysis Journal of Chemical Information and Computer Sciences 43 1 324 336 10.1021/ci0200321 12546568

[b33-tjb-50-01-37] Karali N Gürsoy A Kandemirli F Shvets N Kaynak FB 2007 Synthesis and structure-antituberculosis activity relationship of 1H-indole-2,3-dione derivatives Bioorganic & Medicinal Chemistry 15 17 5888 5904 10.1016/j.bmc.2007.05.063 17561405

[b34-tjb-50-01-37] Karrouchi K Radi S Ramli Y Taoufik J Mabkhot YN 2018 Synthesis and pharmacological activities of pyrazole derivatives: A review Molecules 23 1 134 10.3390/molecules23010134 29329257 PMC6017056

[b35-tjb-50-01-37] Kim JH Jeong JH 2022 Structure-activity relationship studies based on 3D-QSAR CoMFA/CoMSIA for thieno-pyrimidine derivatives as triple negative breast cancer inhibitors Molecules 27 22 7974 10.3390/molecules27227974 36432075 PMC9698756

[b36-tjb-50-01-37] Koirala M Yan L Mohamed Z DiPaola M 2025 AI-integrated QSAR modeling for enhanced drug discovery: From classical approaches to deep learning and structural insight International Journal of Molecular Sciences 26 19 9384 10.3390/ijms26199384 41096653 PMC12525248

[b37-tjb-50-01-37] Krasowski MD Hong X Hopfinger AJ Harrison NL 2002 4D-QSAR analysis of a set of propofol analogues: Mapping binding sites for an anesthetic phenol on the GABA(A) receptor Journal of Medicinal Chemistry 45 15 3210 3221 10.1021/jm010461a 12109905 PMC2864546

[b38-tjb-50-01-37] Li G Cheng Y Han C Song C Huang N 2022 Pyrazole-containing pharmaceuticals: target, pharmacological activity, and their SAR studies RSC Medicinal Chemistry 13 1300 1321 10.1039/D2MD00206J 36439976 PMC9667768

[b39-tjb-50-01-37] Li J Zhao T Yang Q Du S Xu L 2025 A review of quantitative structure-activity relationship: the development and current status of data sets, molecular descriptors and mathematical models Chemometrics and Intelligent Laboratory Systems 256 105278 10.1016/j.chemolab.2024.105278

[b40-tjb-50-01-37] Liang W Ma S Zhang Q Zhu T 2021 Integrative sparse partial least squares Statistics in Medicine 40 9 2239 2256 10.1002/sim.8900 33559203 PMC8071349

[b41-tjb-50-01-37] Librando V Alparone A 2009 The role of electronic properties to the mutagenic activity of 1,6- and 3,6-dinitrobenzo[a]pyrene isomers Journal of Hazardous Materials 161 2–3 1338 1346 10.1016/j.jhazmat.2008.04.095 18571843

[b42-tjb-50-01-37] Luo J Zou H Guo Y Tong T Ye L 2022 SRC kinase-mediated signaling pathways and targeted therapies in breast cancer Breast Cancer Research 24 1 99 10.1186/s13058-022-01596-y 36581908 PMC9798727

[b43-tjb-50-01-37] Maattallaoui I Sakho M Maatallaoui A Catalán EB El Aouad N 2025 Development of QSAR models and web applications for predicting hDHFR inhibitor bioactivity using machine learning Molecules 30 23 4618 10.3390/molecules30234618 41375213 PMC12693464

[b44-tjb-50-01-37] Makkouk AH Bersuker IB Boggs JE 2004 Quantitative Drug Activity Prediction for Inhibitors of Human Breast Carcinoma International Journal of Pharmaceutical Medicine 18 2 81 89

[b45-tjb-50-01-37] Marenich AV Yong PH Bersuker IB Boggs JE 2008 Quantitative antidiabetic activity prediction for the class of guanidino- and aminoguanidinopropionic acid analogs based on electron-conformational studies Journal of Chemical Information and Modeling 48 3 556 568 10.1021/ci700401p 18278893

[b46-tjb-50-01-37] Martins JP Barbosa EG Pasqualoto KFM Ferreira MMC 2009 LQTA-QSAR: A new 4D-QSAR methodology Journal of Chemical Information and Modeling 49 6 1428 1436 10.1021/ci900014f 19422246

[b47-tjb-50-01-37] Melagraki G Afantitis A Makridima K Sarimveis H Igglessi-Markopoulou O 2006 Prediction of toxicity using a novel RBF neural network training methodology Journal of Molecular Modeling 12 3 297 305 https://doi:10.1007/s00894-005-0032-8 16283121 10.1007/s00894-005-0032-8

[b48-tjb-50-01-37] Mohamed HHM Hussien ABWEM Saeed AEM 2022 QSAR and docking studies of pyrazole analogs as antiproliferative against human colorectal adenocarcinoma cell line HT-29 European Journal of Chemistry 13 3 319 326 10.5155/eurjchem.13.3.319-326.2259

[b49-tjb-50-01-37] Muratov EN Bajorath J Sheridan RP Tetko IV Filimonov D 2020 QSAR without borders Chemical Society Reviews 49 11 3525 3564 10.1039/D0CS00098A 32356548 PMC8008490

[b50-tjb-50-01-37] Niazi SK Mariam Z 2023 Recent advances in machine-learning-based chemoinformatics: A comprehensive review International Journal of Molecular Sciences 24 14 11488 10.3390/ijms241411488 37511247 PMC10380192

[b51-tjb-50-01-37] Noronha G Barrett K Boccia A Brodhag T Cao J 2007 Discovery of [7-(2,6-dichlorophenyl)-5-methylbenzo[1,2,4]triazin-3-yl]-[4-(2-pyrrolidin-1-ylethoxy)phenyl]amine — a potent, orally active Src kinase inhibitor with anti-tumor activity in preclinical assays Bioorganic & Medicinal Chemistry Letters 17 3 602 608 10.1016/j.bmcl.2006.11.006 17113292

[b52-tjb-50-01-37] Oprea TI Waller CL Marshall GR 1994 Three-dimensional quantitative structure-activity relationship of human immunodeficiency virus (I) protease inhibitors. 2. Predictive power using limited exploration of alternate binding modes Journal of Medicinal Chemistry 37 14 2206 2215 10.1021/jm00040a013 8035428

[b53-tjb-50-01-37] Padmanabhan J Parthasarathi R Elango M Subramanian V Krishnamoorthy BS 2007 Multiphilic descriptor for chemical reactivity and selectivity Journal of Physical Chemistry A 111 37 9130 9138 10.1021/jp0718909 17715901

[b54-tjb-50-01-37] Patel PK Bhatt HG 2021 Improved 3D-QSAR prediction by multiple conformational alignments and molecular docking studies to design and discover HIV-1 protease inhibitors Current HIV Research 19 2 154 171 10.2174/1570162X18666201119143457 33213349

[b55-tjb-50-01-37] Pavlov T Todorov M Stoyanova G Schmieder P Aladjov H 2007 Conformational coverage by a genetic algorithm: Saturation of conformational space Journal of Chemical Information and Modeling 47 3 851 863 10.1021/ci700014h 17465523

[b56-tjb-50-01-37] Revard BC Tipton WW Hennig RG 2014 Structure and stability prediction of compounds with evolutionary algorithms Topics in Current Chemistry 345 181 222 10.1007/128_2013_489 24515753

[b57-tjb-50-01-37] Rodríguez-Pérez R Miljković F Bajorath J 2022 Machine learning in chemoinformatics and medicinal chemistry Annual Review of Biomedical Data Science 5 43 65 10.1146/annurev-biodatasci-122120-124216 35440144

[b58-tjb-50-01-37] Romeiro NC Albuquerque MG de Alencastro RB Ravi M Hopfinger AJ 2005 Construction of 4D-QSAR models for use in the design of novel p38-MAPK inhibitors Journal of Computer-Aided Molecular Design 19 6 385 400 10.1007/s10822-005-7927-4 16231199

[b59-tjb-50-01-37] Rosines E Bersuker IB Boggs JE 2001 Pharmacophore Identification and Bioactivity Prediction for Group I Metabotropic Glutamate Receptor Agonists by the Electron-Conformational QSAR Method Quantitative Structure-Activity Relationships 20 4 327 334 10.1002/1521-3838(200111)20:4<327::AID-QSAR327>3.0.CO;2-Q

[b60-tjb-50-01-37] Şahin K Saripinar E Yanmaz E Geçen N 2011 Quantitative bioactivity prediction and pharmacophore identification for benzotriazine derivatives using the electron conformational-genetic algorithm in QSAR SAR and QSAR in Environmental Research 22 3–4 217 238 10.1080/1062936X.2010.548341 21391137

[b61-tjb-50-01-37] Sahin K Saripinar E 2020 A novel hybrid method named electron conformational genetic algorithm as a 4D-QSAR investigation to calculate the biological activity of the tetrahydrodibenzazosines Journal of Computational Chemistry 41 1091 1104 10.1002/jcc.26154 32058616

[b62-tjb-50-01-37] Sahin K Saripinar E Durdagi S 2021 Combined 4D-QSAR and target-based approaches for the determination of bioactive Isatin derivatives SAR and QSAR in Environmental Research 32 10 769 792 10.1080/1062936X.2021.1971760 34530651

[b63-tjb-50-01-37] Santos-Filho OA Hopfinger AJ 2006 Structure-based QSAR analysis of a set of 4-hydroxy-5,6-dihydropyrones as inhibitors of HIV-1 protease: an application of the receptor-dependent (RD) 4D-QSAR formalism Journal of Chemical Information and Modeling 46 1 345 354 10.1021/ci050326x 16426069

[b64-tjb-50-01-37] Saripinar E Güzel Y Patat S Yildirim I Akçamur Y 1996 Electron-topological investigation of the structure-antitubercular activity relationship of thiosemicarbazone derivatives Arzneimittel-Forschung/Drug Research 46 8 824 828 9125287

[b65-tjb-50-01-37] Schüürmann G Ebert RU Chen J Wang B Kühne R 2008 External validation and prediction employing the predictive squared correlation coefficient — test set activity mean vs training set activity mean Journal of Chemical Information and Modeling 48 11 2140 2145 10.1021/ci800253u 18954136

[b66-tjb-50-01-37] Seidel T Wieder O Garon A Langer T 2020 Applications of the Pharmacophore Concept in Natural Product inspired Drug Design Molecular Informatics 39 11 e2000059 10.1002/minf.202000059 32578959 PMC7685156

[b67-tjb-50-01-37] Son WJ Jang S Shin S 2012 Simulated Q-annealing: Conformational search with an effective potential Journal of Molecular Modeling 18 1 213 220 10.1007/s00894-011-1072-x 21523533

[b68-tjb-50-01-37] Tao ZF Li G Tong Y Stewart KD Chen Z 2007 Discovery of 4′-(1,4-dihydro-indeno[1,2-c]pyrazol-3-yl)-benzonitriles and 4′-(1,4-dihydro-indeno[1,2-c]pyrazol-3-yl)-pyridine-2′-carbonitriles as potent checkpoint kinase 1 (Chk1 inhibitors Bioorganic & Medicinal Chemistry Letters 17 21 5944 5951 10.1016/j.bmcl.2007.07.102 17827013

[b69-tjb-50-01-37] Todeschini R Consonni V 2000 Handbook of Molecular Descriptors Weinheim Wiley-VCH

[b70-tjb-50-01-37] Tokarski JS Hopfinger AJ 1997 Prediction of ligand-receptor binding thermodynamics by free energy force field (FEFF) 3D-QSAR analysis: application to a set of peptidomimetic renin inhibitors Journal of Chemical Information and Computer Sciences 37 4 792 811 10.1021/ci970006g 9254912

[b71-tjb-50-01-37] Van Damme S Bultinck P 2007 A new computer program for QSAR-analysis: ARTE-QSAR Journal of Computational Chemistry 28 11 1924 1928 10.1002/jcc.20664 17394240

[b72-tjb-50-01-37] Venkatraman V Dalby AR Yang ZR 2004 Evaluation of mutual information and genetic programming for feature selection in QSAR Journal of Chemical Information and Computer Sciences 44 5 1686 1692 10.1021/ci049933v 15446827

[b73-tjb-50-01-37] Xu Y Yao H Lin K 2018 An overview of neural networks for drug discovery and the inputs used Expert Opinion on Drug Discovery 13 12 1091 1102 10.1080/17460441.2018.1547278 30449189

[b74-tjb-50-01-37] Zhai Y Yang J Zhang J Yang J Li Q 2021 Src-family protein tyrosine kinases: A promising target for treating cardiovascular diseases International Journal of Medical Sciences 18 5 1216 1224 10.7150/ijms.49241 33526983 PMC7847615

[b75-tjb-50-01-37] Zhang B Kilburg D Eastman P Pande VS Gallicchio E 2017 Efficient Gaussian density formulation of volume and surface areas of macromolecules on graphical processing units Journal of Computational Chemistry 38 10 740 752 10.1002/jcc.24745 28160511

